# Supplementary figures and images for: Impact of the pandemic and concomitant COVID-19 on the management and outcomes of middle cerebral artery strokes: a nationwide registry-based study
Source: BMJ Open. 2024 Feb 27;14(2):e080738. doi: 10.1136/bmjopen-2023-080738 (PMC10900352; doi:10.1136/bmjopen-2023-080738)

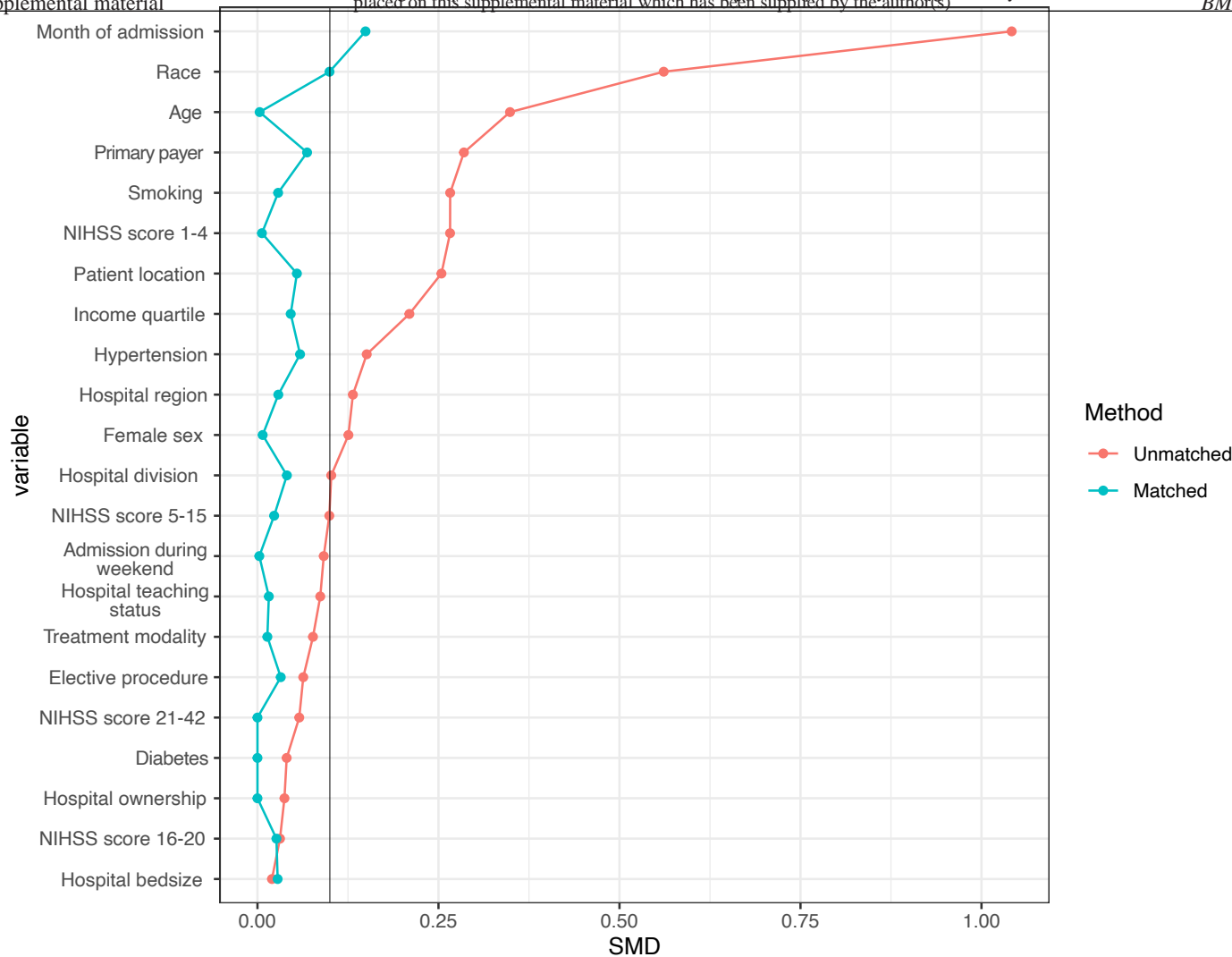

Supplement: Supplementary data [file bmjopen-2023-080738supp001.pdf]

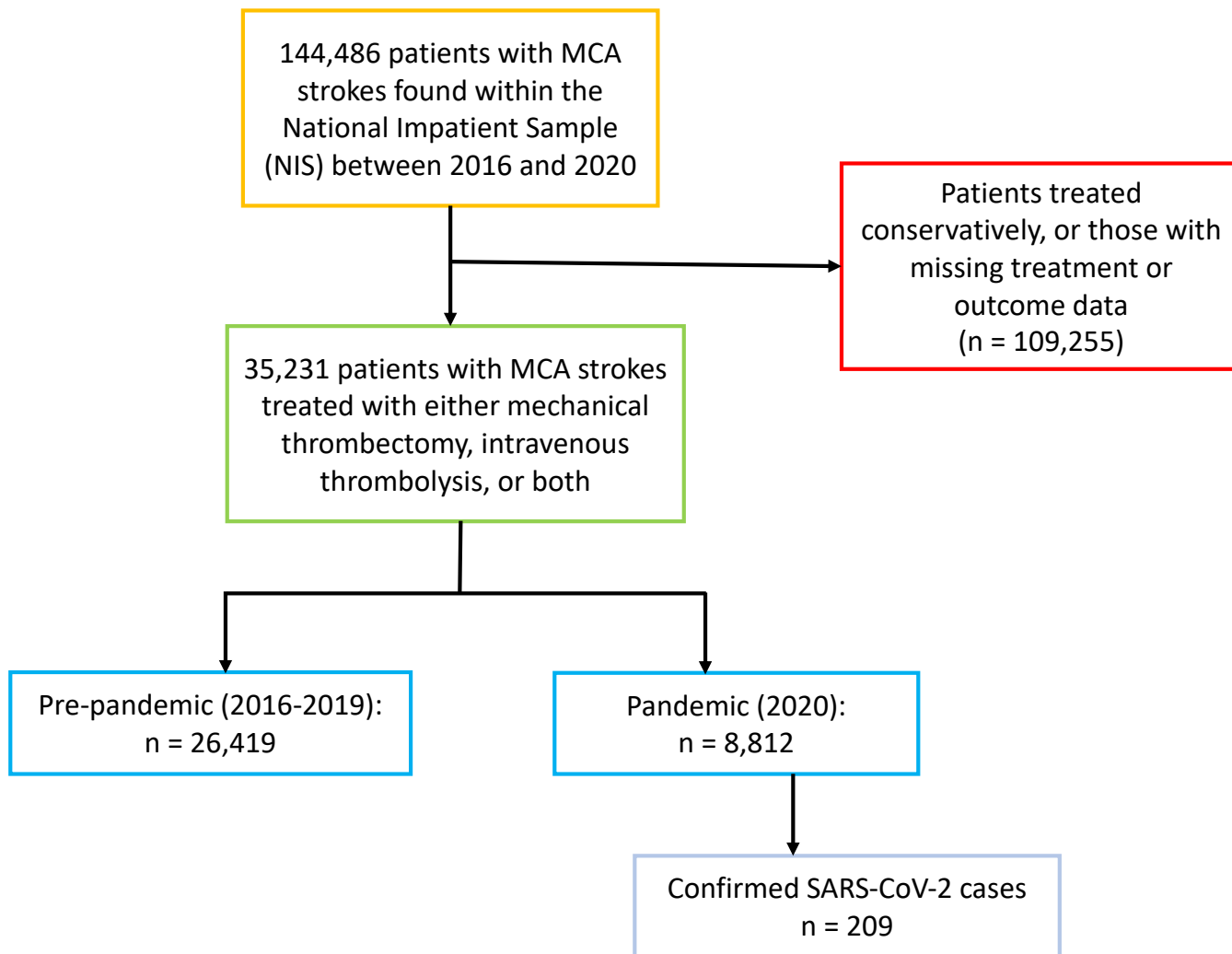

Supplement: Supplementary data [file bmjopen-2023-080738supp002.pdf]
