# Supplementary material for: Impact of the pandemic and concomitant COVID-19 on the management and outcomes of middle cerebral artery strokes: a nationwide registry-based study
Source: BMJ Open. 2024 Feb 27;14(2):e080738. doi: 10.1136/bmjopen-2023-080738 (PMC10900352; doi:10.1136/bmjopen-2023-080738)
Supplement: Author's manuscript [file bmjopen-2023-080738.draft_revisions.pdf]

BMJ Open

BMJ Open is committed to open peer review. As part of this commitment we make the peer review history of every article we publish publicly available.

When an article is published we post the peer reviewers' comments and the authors' responses online. We also post the versions of the paper that were used during peer review. These are the versions that the peer review comments apply to.

The versions of the paper that follow are the versions that were submitted during the peer review process. They are not the versions of record or the final published versions. They should not be cited or distributed as the published version of this manuscript.

BMJ Open is an open access journal and the full, final, typeset and author-corrected version of record of the manuscript is available on our site with no access controls, subscription charges or pay-per-view fees (http://bmjopen.bmj.com).

If you have any questions on BMJ Open's open peer review process please email info.bmjopen@bmj.com

Impact of the Pandemic and Concomitant COVID-19 on the Management and Outcomes of Middle Cerebral Artery Strokes, a Nationwide Analysis

Journal:	*BMJ Open*
Manuscript ID	bmjopen-2023-080738
Article Type:	Original research
Date Submitted by the Author:	09-Oct-2023
Complete List of Authors:	Ghaith, Abdul Karim; Mayo Clinic<br>El-Hajj, Victor Gabriel; Karolinska Institute, Clinical Neuroscience<br>Atallah, Elias; Thomas Jefferson University Hospital, Neurological Surgery<br>Rios Zermeno, Jorge; Mayo Clinic in Florida<br>Ravindran, Krishnan; Mayo Clinic in Florida<br>Gharios, Maria; Karolinska Institute, Clinical Neuroscience<br>Hoang, Harry; Mayo Clinic<br>Bydon, Mohamad; Mayo Clinic<br>Ohlsson, Marcus; Karolinska Institute, Clinical Neuroscience<br>Elmi-Terander, Adrian; Karolinska Institute, Clinical Neuroscience<br>Rabih, Tawk; Mayo Clinic in Florida<br>Jabbour, Pascal; Thomas Jefferson Univ Hosp, Neurosurgery
Keywords:	COVID-19, Stroke < NEUROLOGY, NEUROLOGY

SCHOLARONE™ Manuscripts

BMJ

I, the Submitting Author has the right to grant and does grant on behalf of all authors of the Work (as defined in the below author licence), an exclusive licence and/or a non-exclusive licence for contributions from authors who are: i) UK Crown employees; ii) where BMJ has agreed a CC-BY licence shall apply, and/or iii) in accordance with the terms applicable for US Federal Government officers or employees acting as part of their official duties; on a worldwide, perpetual, irrevocable, royalty-free basis to BMJ Publishing Group Ltd ("BMJ") its licensees and where the relevant Journal is co-owned by BMJ to the co-owners of the Journal, to publish the Work in this journal and any other BMJ products and to exploit all rights, as set out in our licence.

The Submitting Author accepts and understands that any supply made under these terms is made by BMJ to the Submitting Author unless you are acting as an employee on behalf of your employer or a postgraduate student of an affiliated institution which is paying any applicable article publishing charge ("APC") for Open Access articles. Where the Submitting Author wishes to make the Work available on an Open Access basis (and intends to pay the relevant APC), the terms of reuse of such Open Access shall be governed by a Creative Commons licence – details of these licences and which Creative Commons licence will apply to this Work are set out in our licence referred to above.

Other than as permitted in any relevant BMJ Author's Self Archiving Policies, I confirm this Work has not been accepted for publication elsewhere, is not being considered for publication elsewhere and does not duplicate material already published. I confirm all authors consent to publication of this Work and authorise the granting of this licence.

For peer review only

Impact of the Pandemic and Concomitant COVID-19 on the Management and Outcomes of Middle Cerebral Artery Strokes, a Nationwide Analysis

Authors: Abdul Karim Ghaith MD [1,*]; Victor Gabriel El-Hajj [1,2,*]; Elias Atallah MD [3]; Jorge Rios-Zermeno MD [4]; Krishnan Ravindran MD [4]; Maria Gharios BS [2]; Harry Hoang [1]; Mohamad Bydon MD [1]; Marcus Ohlsson MD, PhD [2]; Adrian Elmi-Terander MD, PhD [2]; Rabih Tawk MD [4]; Pascal Jabbour MD [3]

[1]Department of Neurological Surgery, Mayo Clinic, Rochester, Minnesota, USA.
[2]Department of Clinical Neuroscience, Karolinska Institutet, Stockholm, Sweden.
[3]Department of Neurological Surgery, Thomas Jefferson University Hospital, Philadelphia, USA
[4]Department of Neurological Surgery, Mayo Clinic, Jacksonville, Florida, USA.
[5]Department of Surgical Sciences, Uppsala University, Uppsala, Sweden
[6]Capio Spine Center Stockholm, Löwenströmska Hospital, Upplands Väsby, Sweden
*Equal contributions

Corresponding author

Name: Adrian Elmi-Terander

Email: adrian.elmi.terander@stud.ki.se

Adress: Capio Spine Center Stockholm, Löwenströmska Hospital, 194 02 Upplands-Väsby, Sweden (Box 2074)

Telephone number: +46704716766

For peer review only

Abstract

Introduction

Stroke presentations significantly dropped during the COVID-19 pandemic, owing to global alterations in healthcare seeking behaviors as well as re-allocation of healthcare resources. However, infection with the virus itself has clearly been linked with an increased risk of stroke.

Objective

To investigate the impact of COVID-19 on stroke care, focusing on middle cerebral artery (MCA) territory infarctions.

Methods

A National Inpatient Sample (NIS) based study was conducted on patients with MCA strokes treated between 2016 and 2020. Primary outcomes were postprocedural complications, length of stays, in-hospital mortality, and non-routine discharge. Propensity score matching was performed to reduce confounders when comparing groups.

Results

In total, 35,231 patients with MCA strokes were included. Mechanical thrombectomy (MT) was performed in 48.4% of patients, while 38.2% received intravenous thrombolysis (IVT), and 13.4% received both mechanical thrombectomy and IVT (MT-IVT). A gradual increase in the use of MT and an opposite decrease in the use of IVT ($p<0.001$) was detected during the study period. Overall, 25.0% of all patients were admitted for MCA strokes during the pandemic (2020), of these 209 (2.4%) were concomitantly diagnosed with COVID-19. Patients with MCA strokes and concomitant COVID-19 were significantly younger (64.9 vs. 70.0; $p<0.001$), had significantly worse NIHSS scores, and outcomes in terms of length of stays ($p<0.001$), in-hospital mortality ($p<0.001$), and non-routine discharges ($p=0.013$), as compared to those without COVID-19. After matching, only in-hospital mortality remained significantly worse among patients with COVID-19 ($p<0.001$).

Conclusions

For peer review only

Among patients with MCA stroke, those with concomitant COVID-19 were significantly younger and had higher stroke severity scores on the NIHSS scale. They were more likely to experience thromboembolic complications and in-hospital mortality compared to matched controls.

Data availability statement

Data are available upon reasonable request.

For peer review only

Strengths and limitations

- This study includes more than 35,000 patients admitted for strokes of the middle cerebral artery (MCA) between 2016 and 2020
- Our study attempted to discern the impact of the pandemic state itself from that of concomitant infection with SARS-CoV-2 on the management and outcomes of MCA strokes.
- This study is registry-based and limited by its retrospective and hospital-based rather than population-based nature.

Introduction

Cerebral stroke is a leading cause of morbidity and mortality. In 2019 stroke was globally ranked second as the leading cause of death[1] and disability-adjusted life years (DALYs) in individuals aged above 50.[2] During that year, 12.2 million new cases of stroke were reported in over 204 countries and territories. Stroke was responsible for 6.55 million deaths, where hemorrhagic stroke was slightly overrepresented compared to ischemic.[1] Recent estimates suggest that one of every four individuals are at risk of experiencing a stroke during their lifetime.[3] In addition to the associated mortality and morbidity, stroke is known to generate a significant financial burden on both individual and societal levels. In the US, the costs of stroke add up to $59,900 per patient and year.[4,5,6]

The pandemic caused by SARS-CoV-2 (COVID-19 pandemic) hitting the world by the end of 2019, was set to impact the healthcare infrastructure in an unprecedented fashion. The COVID-19 pandemic has, directly and indirectly, caused major epidemiological changes to cerebrovascular diseases.[7] While stroke presentations significantly dropped during the pandemic,[8,9] infection with the virus was linked with an increased risk of strokes .[10–13] The pathophysiology behind this association is thought to be a hypercoagulable state, in turn, linked to abnormal platelet activation, endothelial dysfunction, and disruption of the coagulation cascade by the virus.[7,14] Other noteworthy changes during the pandemic were the rise in the prevalence of younger individuals and those with large vessel obstructions among patients with stroke.[15,16] Apart from changes to the epidemiology of stroke, several reports have identified alterations in the use of different treatment modalities during that time.[15,17,18] While many of these changes have been hypothesized to result from altered signaling pathways associated with infection with the virus, altered care-seeking behaviors, and the burden of the pandemic on healthcare infrastructures may also have played a role.[7] However, few studies on the impact of concomitant SARS-CoV-2 infection on the management and outcomes of stroke have been conducted. Using data provided by the NIS, the aim of this study was to validate previous reports and investigate the effects of the COVID-19 pandemic and SARS-CoV-2 infection on stroke care and short-term outcomes, focusing on middle cerebral artery (MCA) territory infarctions (Figure 1).[19]

For peer review only

Methods

Data Source

The NIS, which is maintained by the Healthcare Cost and Utilization Project, is one of the largest inpatient care databases accessible to the public in the United States. The dataset includes approximately 7 million unweighted patient records per year, representing a 20% stratified sample of all Healthcare Cost and Utilization Project community hospitals in the United States. The NIS permits extensive investigations into healthcare utilization, access, charges, quality, and outcomes and provides dependable national estimates on an annual basis. The data contains a variety of elements, including demographic characteristics, hospital and regional information, diagnoses, procedures, and discharge disposition for all patients for whom documentation exists. More information about the NIS is available at www.hcup-us.ahrq.gov. As the data was collected from a de-identified national database, Institutional Review Board (IRB) approval was not required.

Patient and public involvement

Patients were not involved in the design or conception of the study.

Cohort selection

Patients diagnosed and treated with ischemic middle cerebral artery (MCA) stoke were identified using the International Classification of Diseases (ICD) codes “I66.0”, “I66.01”, “I66.02”, “I66.03”, “I66.09”, “I63.31”, “I633.11”, “I633.13”,“I63.319”, “I63.411”, “I63.412”, “I63.413”, “I63.419”, “I63.51”. “I63.512”, “I63.513”, “I63.513”, “I63.519” from the 10th revisions. The study considered three primary treatment modalities: mechanical thrombectomy (MT), intravenous thrombolysis (IVT), or combination of them (MT+IVT). MCA stroke patients that received no treatment were excluded from the study. Initially, 144,486 patients with MCA strokes were identified within the NIS database between 2016 to 2020. However, 64,422 patients were excluded as their treatment approach was not specified, leaving 35,231 patients for inclusion in this study. The study was performed in accordance with the STROBE guidelines.

Outcomes and variables

For peer review only

The following variables were extracted: age, gender, race, insurance type, smoking status, history of smoking, hypertension, diabetes, and stroke severity measured by NIH stroke severity (NIHSS) score. Hospital-related data were recorded, including household income quartile, bed size, location (rural/urban), teaching status, region (Northeast, Midwest, South, West), and control/ownership of the hospital.

The study examined several key outcomes:

Death, non-routine discharge (transfer to short-term hospital, skilled nursing or intermediate care facility, home health care, or against medical advice), length of stay (LOS in days).

Intervention-related complications such as access-site hemorrhage, subarachnoid hemorrhages (SAH), vasospasm, intracerebral or intraventricular hemorrhage.

Medical complications including neurological, cardiac, pulmonary, urinary, and thromboembolic events (deep venous thrombosis DVT and pulmonary embolisms PE).

Propensity score matching and statistical analysis

Using the Mann-Kendall test, trends regarding the yearly incidence of MCA ischemic stroke cases, treatment modalities, and postprocedural complications between 2016 and 2020 were evaluated.[20] When analyzing the impact of concomitant COVID-19 on management and outcomes of MCA strokes, 4:1 propensity score matching based on all available baseline variables, featured in the love plot (Supplementary Figure A), was performed, using the K-nearest method with a caliper of 0.2. All statistical analyses were conducted using Python and R software.[21]

For peer review only

Results

Trend analysis

A total of 35,231 patients were included (Supplementary Figure B), with 13,465 receiving IVT, 17,060 undergoing mechanical thrombectomy, and 4,706 undergoing mechanical thrombectomy and IVT. Results from the trend analysis revealed that the number of patients with MCA stroke within the NIS database significantly increased between the years 2016 and 2020 ($p = 0.028$). The proportion of patients receiving IVT decreased significantly ($p = 0.027$) due to a gradual and significant increase in MT ($p = 0.027$). There were no changes in the proportion of patients undergoing both IVT and MT ($p = 0.086$; Figure 2). The complications rates remained stable ($p > 0.05$), except for a slight increase in postprocedural SAHs ($p = 0.043$), thromboembolic events ($p = 0.043$), and urinary tract infections ($p = 0.027$; Figures 3A and 3B).

Impact of the pandemic on management of patients with MCA stroke

There were 8,291 patients admitted for MCA stroke the year before the pandemic (2019) and 8,812 during the pandemic (2020) (Table 1). During 2020, 209 of the patients (2.4%) were concomitantly infected by SARS-CoV-2. There were no differences with respect to demographics, including age, sex, race or ethnicity, primary payer, and hospital location among the two admission periods. During the pandemic, urban, non-teaching hospitals received a larger share of patients with MCA stroke, compared to the year before (9.7% vs. 8.5%; $p = 0.012$). The choice of treatment modalities significantly differed between the two admission periods, with MT being more commonly performed during than prior to the pandemic, ($p = 0.005$). The length of hospital stay was similar between the two time periods (mean: 8.2 ± 10.0; $p = 0.239$). Postprocedural complications remained similarly prevalent r, with subarachnoid hemorrhages occurring in 5.8% of patients, vasospasm in 0.9%, thromboembolic events in 5.3%, neurological complications in 0.5%, and cardiac complications in 0.5% of patients ($p > 0.05$). However, access site hemorrhage was more common during the pre-pandemic period (1.2% vs. 0.9%; $p = 0.037$), while acute kidney injury (18.5% vs. 16.4%; $p < 0.001$), respiratory (6.7% vs. 5.9%; $p = 0.030$), and urinary complications (18.5% vs. 16.4%; $p < 0.001$) were more common during the the pandemic, .

For peer review only - http://bmjopen.bmj.com/site/about/guidelines.xhtml

While the proportion of non-routine discharge (76.6%) did not significantly differ between admission periods ($p = 0.149$), there were significantly more in-hospital deaths among patients admitted for MCA stroke during the pandemic (10.2% vs. 9.0%; $p = 0.010$) (Table 1).

For peer review only

Table 1. Differences between patients admitted for MCA strokes before and during the pandemic (2019 vs. 2020).

	**Total (N=17103)**	**Pre-pandemic (N=8291)**	**During the pandemic (N=8812)**	**P-value**
Female	8825 (51.6%)	4322 (52.1%)	4503 (51.1%)	0.176
Mean Age (SD)	70.0 (14.6)	70.2 (14.6)	69.8 (14.6)	0.141
Race and ethnicity				0.124
White	11315 (66.2%)	5545 (66.9%)	5770 (65.5%)	
Black	2602 (15.2%)	1232 (14.9%)	1370 (15.5%)	
Hispanic	1391 (8.1%)	657 (7.9%)	734 (8.3%)	
Asian or Pacific Islander	566 (3.3%)	286 (3.4%)	280 (3.2%)	
Native American	59 (0.3%)	32 (0.4%)	27 (0.3%)	
Other	557 (3.3%)	269 (3.2%)	288 (3.3%)	
SARS-CoV-2 positive	209 (1.2%)	n/a	209 (2.4%)	n/a
Income quartile				**0.005**
1	4718 (27.6%)	2295 (27.7%)	2423 (27.5%)	
2	4336 (25.4%)	2014 (24.3%)	2322 (26.4%)	
3	4209 (24.6%)	2130 (25.7%)	2079 (23.6%)	
4	3570 (20.9%)	1722 (20.8%)	1848 (21.0%)	
Primary payer				0.652
Medicare	10822 (63.3%)	5267 (63.5%)	5555 (63.0%)	
Medicaid	1659 (9.7%)	772 (9.3%)	887 (10.1%)	
Private insurance	3501 (20.5%)	1715 (20.7%)	1786 (20.3%)	
Self-pay	648 (3.8%)	318 (3.8%)	330 (3.7%)	
No charge	51 (0.3%)	24 (0.3%)	27 (0.3%)	
Other	398 (2.3%)	183 (2.2%)	215 (2.4%)	
Hospital region				0.439
Northeast	2975 (17.4%)	1441 (17.4%)	1534 (17.4%)	
Midwest	3496 (20.4%)	1696 (20.5%)	1800 (20.4%)	
South	6974 (40.8%)	3421 (41.3%)	3553 (40.3%)	
West	3658 (21.4%)	1733 (20.9%)	1925 (21.8%)	
Hospital location and teaching status				**0.012**
Rural	207 (1.2%)	109 (1.3%)	98 (1.1%)	
Urban non-teaching	1555 (9.1%)	702 (8.5%)	853 (9.7%)	
Urban teaching	15341 (89.7%)	7480 (90.2%)	7861 (89.2%)	

For peer review only

	**Total (N=17103)**	**Pre-pandemic (N=8291)**	**During the pandemic (N=8812)**	**P-value**
Hospital bed size				0.746
Small	1560 (9.1%)	754 (9.1%)	806 (9.1%)	
Medium	3983 (23.3%)	1952 (23.5%)	2031 (23.0%)	
Large	11560 (67.6%)	5585 (67.4%)	5975 (67.8%)	
Treatment modality				**0.005**
IVT	5693 (33.3%)	2855 (34.4%)	2838 (32.2%)	
MT	9089 (53.1%)	4309 (52.0%)	4780 (54.2%)	
MT with IVT	2321 (13.6%)	1127 (13.6%)	1194 (13.5%)	
**Outcomes**				
Mean length of stay (LOS) in days (SD)	8.2 (10.0)	8.1 (9.2)	8.3 (10.8)	0.239
Subarachnoid hemorrhage	993 (5.8%)	472 (5.7%)	521 (5.9%)	0.540
Intracranial hemorrhage	2945 (17.2%)	1422 (17.2%)	1523 (17.3%)	0.819
Intraventricular hemorrhage	270 (1.6%)	128 (1.5%)	142 (1.6%)	0.723
Vasospasm	150 (0.9%)	70 (0.8%)	80 (0.9%)	0.656
Access site hemorrhage	174 (1.0%)	98 (1.2%)	76 (0.9%)	**0.037**
Hematoma	74 (0.4%)	35 (0.4%)	39 (0.4%)	0.839
Wound dehiscence	15 (0.1%)	5 (0.1%)	10 (0.1%)	0.240
Vascular catheter infection	12 (0.1%)	7 (0.1%)	5 (0.1%)	0.494
Thromboembolic events	901 (5.3%)	424 (5.1%)	477 (5.4%)	0.382
Acute kidney injury	2986 (17.5%)	1360 (16.4%)	1626 (18.5%)	< **0.001**
Neurologic complications	94 (0.5%)	48 (0.6%)	46 (0.5%)	0.615
Respiratory complications	1084 (6.3%)	491 (5.9%)	593 (6.7%)	**0.030**
Cardiac complications	92 (0.5%)	43 (0.5%)	49 (0.6%)	0.738
Urinary complications	2986 (17.5%)	1360 (16.4%)	1626 (18.5%)	< **0.001**

For peer review only

	**Total (N=17103)**	**Pre-pandemic (N=8291)**	**During the pandemic (N=8812)**	**P-value**
Non-routine discharge	13099 (76.6%)	6310 (76.1%)	6789 (77.1%)	0.149
In-hospital mortality	1646 (9.6%)	748 (9.0%)	898 (10.2%)	**0.010**

For peer review only

Impact of concomitant COVID-19 on management and short-term outcomes of MCA stroke

Prior to the propensity score matched analysis, 209 patients with COVID-19 (SARS-CoV-2 positive) were compared to 8,603 without COVID-19, admitted during 2020 (Table 2). A map showing the distribution of patients with MCA stroke and concomitant COVID-19 is presented (Figure 4). Patients with COVID-19 tended to be males (55% vs. 48.8%; $p = 0.073$) and were significantly younger (64.9 ± 14.4 vs. 70.0 ± 14.6; $p < 0.001$). White patients with MCA stroke were significantly less likely to present with concomitant COVID-19, opposed to all other races and ethnicities ($p < 0.001$). COVID-19 patients tended to cluster within lower income quartiles ($p = 0.065$) and were significantly more covered by Medicaid (16.35 vs. 9.9%; $p < 0.001$). There were no significant differences in hospital region, location, teaching status or number of beds ($p > 0.05$). Patients with COVID-19 had significantly worse NIHSS scores (16-42) ($p = 0.015$). The treatment of choice did not significantly differ between the groups ($p = 0.562$). The mean length of hospital stay was significantly longer among patients with COVID-19 (12.3 ± 12.0 vs. 8.2 ± 10.7; $p < 0.001$). The total hospital charges were significantly higher for patients with COVID-19 ($p < 0.001$). Medical, complications, including thromboembolic events, acute kidney failure, respiratory, and urinary infection were significantly more prevalent amongst patients with COVID-19 ($p < 0.05$). Similarly, non-routine discharges ($p < 0.001$) and in-hospital mortality ($p < 0.001$) were significantly more common in this patient group.

After propensity score matching using patient demographics, baseline characteristics and comorbidities, and stroke severity on the NIHSS scale (Supplementary Figure A), only the rate of thromboembolic events ($p = 0.035$), respiratory complications ($p = 0.029$), and in-hospital mortality ($p < 0.001$) remained significant. This suggests a direct correlation between COVID-19 and the occurrence of complications and as in-hospital mortality. This was further verified using multivariable logistic regression, which indicated that concomitant infection with SARS-CoV-2 was a significant and independent predictor of in-hospital mortality ($p < 0.01$). Surprisingly, COVID-19 patients were seemingly less affected by hemorrhages, in particular intracranial ones, compared to patients without COVID-19 (23.5% vs. 15.4%; $p = 0.014$) (Table 2).

For peer review only

Table 2. Differences between covid positive and negative patients admitted for MCA strokes during the pandemic year of 2020.

	**Pre-matching analysis**			**Propensity score matched analysis**		
	**SARS-CoV-2 positive (N=209)**	**SARS-CoV-2 negative (N=8603)**	**P-value**	**SARS-CoV-2 positive (N=195)**	**SARS-CoV-2 negative (N=753)**	**P-value**
Female sex	94 (45.0%)	4409 (51.2%)	0.073	90 (46.2%)	346 (45.9%)	0.959
Mean age (SD)	64.9 (14.4)	70.0 (14.6)	**< 0.001**	65.4 (14.3)	65.7 (15.7)	0.773
NIHSS score			**0.015**			0.991
1-4	8 (3.8%)	918 (10.7%)		8 (4.1%)	31 (4.1%)	
5-15	66 (31.6%)	3121 (36.3%)		65 (33.3%)	249 (33.1%)	
16-20	34 (16.3%)	1302 (15.1%)		31 (15.9%)	125 (16.6%)	
21-42	42 (20.1%)	1534 (17.8%)		36 (18.5%)	148 (19.7%)	
N-miss	59 (28.2%)	1728 (20.1%)		55 (28.2%)	200 (26.6%)	
Treatment modality			0.562			0.979
IVT	65 (31.1%)	2773 (32.2%)		62 (31.8%)	245 (32.5%)	
MT	120 (57.4%)	4660 (54.2%)		110 (56.4%)	419 (55.6%)	
MT with IVT	24 (11.5%)	1170 (13.6%)		23 (11.8%)	89 (11.8%)	
**Outcomes**						
Mean length of stay (LOS) in days (SD)	12.3 (12.0)	8.2 (10.7)	**< 0.001**	10.6 (8.6)	10.4 (9.0)	0.788
Mean total charges in USD (SD)	255,920 (238,810)	192,269 (183,666)	**< 0.001**	229,414 (193,518)	231,857 (196,519)	0.878
Subarachnoid hemorrhage	16 (7.7%)	505 (5.9%)	0.280	14 (7.2%)	53 (7.0%)	0.945
Intracranial hemorrhage	33 (15.8%)	1490 (17.3%)	0.563	30 (15.4%)	177 (23.5%)	**0.014**
Intraventricular hemorrhage	2 (1.0%)	140 (1.6%)	0.447	2 (1.0%)	14 (1.9%)	0.421
Access site hemorrhage	0 (0.0%)	76 (0.9%)	0.172	0 (0.0%)	3 (0.4%)	0.377
Vasospasm	2 (1.0%)	78 (0.9%)	0.940	2 (1.0%)	11 (1.5%)	0.641
Hemorrhagic stroke	18 (8.6%)	710 (8.3%)	0.852	18 (9.2%)	83 (11.0%)	0.470
Wound dehiscence	1 (0.5%)	9 (0.1%)	0.113	1 (0.5%)	2 (0.3%)	0.584
Vascular catheter infection	0 (0.0%)	5 (0.1%)	0.727	0 (0.0%)	2 (0.3%)	0.471
Thromboembolic events	25 (12.0%)	452 (5.3%)	**< 0.001**	24 (12.3%)	57 (7.6%)	**0.035**

For peer review only

	**Pre-matching analysis**			**Propensity score matched analysis**		
	**SARS-CoV-2 positive (N=209)**	**SARS-CoV-2 negative (N=8603)**	**P-value**	**SARS-CoV-2 positive (N=195)**	**SARS-CoV-2 negative (N=753)**	**P-value**
Acute kidney injury	56 (26.8%)	1570 (18.2%)	**0.002**	49 (25.1%)	152 (20.2%)	0.132
Myocardial infarction	15 (7.2%)	487 (5.7%)	0.350	12 (6.2%)	47 (6.2%)	0.964
Neurologic complications	0 (0.0%)	46 (0.5%)	0.289	0 (0.0%)	7 (0.9%)	0.177
Respiratory complications	26 (12.4%)	567 (6.6%)	**< 0.001**	22 (11.3%)	50 (6.6%)	**0.029**
Cardiac complications	0 (0.0%)	49 (0.6%)	0.274	0 (0.0%)	5 (0.7%)	0.254
Urinary complications	56 (26.8%)	1570 (18.2%)	**0.002**	49 (25.1%)	152 (20.2%)	0.132
Non-routine discharge	176 (84.2%)	6613 (76.9%)	**0.013**	165 (84.6%)	615 (81.8%)	0.355
In-hospital mortality	55 (26.3%)	843 (9.8%)	**< 0.001**	52 (26.7%)	64 (8.5%)	**< 0.001**

For peer review only

Discussion

In this study, 35,231 patients with MCA stroke between 2016 and 2020 were included. Results from the trend analysis revealed that the number of patients with MCA stroke within the NIS database gradually increased during the study period including the pandemic (2020). This is in opposition with previous reports showing a decrease in stroke admissions during the pandemic. Global reports have indicated a decline in the volume of stroke hospitalizations, with primary stroke centers and centers with higher COVID-19 inpatient volumes experiencing steeper declines.[7,8,17] A recovery of initial stroke hospitalization rates was not witnessed until later during the pandemic.[17] The results of this study mainly reflect the coverage of the NIS registry and may merely indicate an expansion of the database during that time, rather than an actual increase in stroke admissions. The proportion of patients receiving IVT significantly decreased during the study period (48% to 32%; p = 0.027), due to a gradual and significant increase in MT (40% to 55%; p = 0.027).

To our knowledge, there are no previous works contrasting the impact of concomitant infection with SARS-CoV-2 with the impact of the unique circumstances created by the pandemic itself on the management of MCA strokes.

In this study, patients with COVID-19 were younger (p<0.001) but had significantly worse NIHSS scores (16-42). A meta-analysis on 129 491 patients reached similar conclusions, revealing that patients who were admitted for stroke, were significantly younger, and had strokes of higher severity grades. There have been several reports highlighting the occurrence of large-vessel occlusions in young patients with COVID-19.[16,22,23] In one study, patients with stroke and concomitant COVID-19 were significantly younger and lacked vascular risk factors. Despite being healthier at baseline, these patients had poorer outcomes and were less likely to experience complete revascularization compared to matched controls without COVID-19.[16] The occurrence of larger stroke in this group of younger and healthier patients was hypothesized to be due to the hypercoagulable state associated with infection with SARS-CoV-2, leading to thrombosis (Figure 5).[7,14,24,25]

Comparing the pre-pandemic year of 2019 with the year of 2020 during which the first wave of the pandemic occurred, we found that MT was more commonly performed than IVT (p = 0.005). Although this discrepancy may be explained by the fact that large vessel occlusions occurred more frequently among patients with COVID-19,[7,15] our results did not reveal any significant difference in treatment modality between patients with and without COVID-19.

For peer review only

Also, a significantly increased risk of thromboembolic events in patients with MCA strokes and concomitant COVID-19, was found in the current study. This highlights the well-established correlation between COVID-19 and the occurrence of thromboembolic events,[26,27] which lead to recommendations for prophylactic treatment of hospitalized COVID-19 patients with anticoagulation therapy.[28] Surprisingly, post-matching results indicated that 23.5% of patients without COVID-19 experienced intracranial hemorrhage compared to 15.4% of those with COVID-19 ($p = 0.014$). We hypothesize that this effect may have been the result of the hypercoagulable state associated with COVID-19.[7,14,24,25] Although previous reports seem to suggest the opposite; with increased rates of intracranial hemorrhage among patients with COVID-19, this association was mainly related to the treatment with anticoagulation therapy in this group of patients.[29] Additionally, the study period only considers the first year of the pandemic, during which recommendations regarding prophylactic anticoagulation therapy still were not generalized. This, may explain the lower hemorrhage risk among these patients.[15]

Lastly, our study revealed a slightly higher in-hospital mortality rate during the first year of the pandemic as compared to the year before that ($p = 0.010$). This may be due to a number of factors. Firstly, the strain on the healthcare systems at that time, may have created disruptions in routine care leading to suboptimal treatment of this patient group. Additionally, the fear of contracting COVID-19 as well as the public health measures undertaken during this period may have led to delayed medical attention for stroke patients. This may have contributed to more severe cases of stroke and in turns a higher mortality. Also, concomitant infection with SARS-CoV-2 itself may have contributed to the increased death toll through larger strokes, as previously mentioned, or an increased rate of adverse events. In fact, longer length of stays ($p < 0.001$), risk of non-routine discharge ($p = 0.013$), and higher in-hospital mortality rate ($p < 0.001$) were all noted among patients with MCA stroke and concomitant COVID-19, which may have been the result of higher stroke severity (NIHSS scores) in this group. After adjusting for confounders, including the NHSS scores through propensity score matching, only in-hospital mortality remained significant ($p < 0.001$). This direct correlation between COVID-19 and in-hospital mortality was likely the result of the increased prevalence of thromboembolic events ($p = 0.035$) and respiratory complications ($p = 0.029$) witnessed in patients with COVID-19.

For peer review only

Limitations

This study has several limitations. Primarily, the sample size of patients with concomitant Covid-19 infection was very small compared to the whole cohort of patients with MCA stroke. The data provided by the NIS is limited by its retrospective and hospital-based rather than population-based nature. Additionally, as with all registry-based studies, there is a risk of reporting and coding biases, loss to follow-up, and attrition, as well as other weaknesses. The NIS also lacks clinically important endpoints, such as patient-reported, health-related quality of life, neurological, and long-term clinical outcomes, as well as granularity in terms of the cause of death and other epidemiological elements. Moreover, the registry only targets the U.S. population which limits international generalizability and calls for external validation of the findings. Although propensity score matching was performed using all baseline data available on hand, the absence of confounding variables, such as various comorbidities, owing to the dataset's retrospective design limited our ability to pinpoint the true effect of COVID-19 on the outcomes of interest.

For peer review only

Conclusion

Among patients with MCA stroke, those with concomitant COVID-19 were significantly younger and had higher stroke severity scores on the NIHSS scale. They were more likely to experience thromboembolic complications and in-hospital mortality compared to matched controls.

For peer review only

Competing interests

No competing interests are reported by any of the authors.

Author Contributions

- AKG, VGE: conception & design of the work, drafting of the article, critical revision, and final approval of the version to be published.
- EA, JRZ, KR, MG, HH: conception & design of the work, drafting of the article, and final approval of the version to be published.
- MB, MO, AET, RT: conception & design of the work, drafting of the article, critical revision, and final approval of the version to be published.
- PJ: guarantor of the review, conception & design of the work, drafting of the article, critical revision, and final approval of the version to be published.

Funding

None of the authors received funding.

Ethics declarations

Ethics approval and consent to participate

This study was performed in accordance with all ethical guidelines. The need for ethical approval was waved by the Mayo IRB due to the deidentified nature of the NIS database. Consent to participate is not applicable.

Consent for publication

Not applicable.

For peer review only

Acknowledgements

Not applicable.

For peer review only

References:

1. GBD 2019 Stroke Collaborators. Global, regional, and national burden of stroke and its risk factors, 1990-2019: a systematic analysis for the Global Burden of Disease Study 2019. *Lancet Neurol*. 2021;20(10):795-820.

2. GBD 2019 Diseases and Injuries Collaborators. Global burden of 369 diseases and injuries in 204 countries and territories, 1990-2019: a systematic analysis for the Global Burden of Disease Study 2019. *Lancet*. 2020;396(10258):1204-1222.

3. GBD 2016 Lifetime Risk of Stroke Collaborators, Feigin VL, Nguyen G, et al. Global, Regional, and Country-Specific Lifetime Risks of Stroke, 1990 and 2016. *N Engl J Med*. 2018;379(25):2429-2437.

4. Strilciuc S, Grad DA, Radu C, et al. The economic burden of stroke: a systematic review of cost of illness studies. *J Med Life*. 2021;14(5):606-619.

5. Ovbiagele B, Goldstein LB, Higashida RT, et al. Forecasting the future of stroke in the United States: a policy statement from the American Heart Association and American Stroke Association. *Stroke*. 2013;44(8):2361-2375.

6. Vermeer SE, Longstreth WT Jr, Koudstaal PJ. Silent brain infarcts: a systematic review. *Lancet Neurol*. 2007;6(7):611-619.

7. Siegler JE, Abdalkader M, Michel P, Nguyen TN. Therapeutic Trends of Cerebrovascular Disease during the COVID-19 Pandemic and Future Perspectives. *J Stroke Cerebrovasc Dis*. 2022;24(2):179-188.

8. Ishaque N, Butt AJ, Kamtchum-Tatuene J, et al. Trends in Stroke Presentations before and during the COVID-19 Pandemic: A Meta-Analysis. *J Stroke Cerebrovasc Dis*. 2022;24(1):65-78.

9. Perry R, Banaras A, Werring DJ, Simister R. What has caused the fall in stroke admissions during the COVID-19 pandemic? *J Neurol*. 2020;267(12):3457-3458.

10. Katsoularis I, Fonseca-Rodríguez O, Farrington P, Lindmark K, Fors Connolly AM. Risk of acute myocardial infarction and ischaemic stroke following COVID-19 in Sweden: a self-controlled case series and matched cohort study. *Lancet*. 2021;398(10300):599-607.

For peer review only

11. Tu TM, Seet CYH, Koh JS, et al. Acute Ischemic Stroke During the Convalescent Phase of Asymptomatic COVID-2019 Infection in Men. *JAMA Netw Open*. 2021;4(4):e217498.

12. Mao L, Jin H, Wang M, et al. Neurologic Manifestations of Hospitalized Patients With Coronavirus Disease 2019 in Wuhan, China. *JAMA Neurol*. 2020;77(6):683-690.

13. Merkler AE, Parikh NS, Mir S, et al. Risk of Ischemic Stroke in Patients With Coronavirus Disease 2019 (COVID-19) vs Patients With Influenza. *JAMA Neurol*. 2020;77(11):1-7.

14. Abou-Ismail MY, Diamond A, Kapoor S, Arafah Y, Nayak L. The hypercoagulable state in COVID-19: Incidence, pathophysiology, and management. *Thromb Res*. 2020;194:101-115.

15. Katsanos AH, Palaiodimou L, Zand R, et al. Changes in Stroke Hospital Care During the COVID-19 Pandemic: A Systematic Review and Meta-Analysis. *Stroke*. 2021;52(11):3651-3660.

16. Jabbour P, Dmytriw AA, Sweid A, et al. Characteristics of a COVID-19 Cohort With Large Vessel Occlusion: A Multicenter International Study. *Neurosurgery*. 2022;90(6):725-733.

17. Nogueira RG, Qureshi MM, Abdalkader M, et al. Global Impact of COVID-19 on Stroke Care and IV Thrombolysis. *Neurology*. 2021;96(23):e2824-e2838.

18. Katsanos AH, Palaiodimou L, Zand R, et al. The Impact of SARS-CoV-2 on Stroke Epidemiology and Care: A Meta-Analysis. *Ann Neurol*. 2021;89(2):380-388.

19. Ng YS, Stein J, Ning M, Black-Schaffer RM. Comparison of clinical characteristics and functional outcomes of ischemic stroke in different vascular territories. *Stroke*. 2007;38(8):2309-2314.

20. Kim HJ, Luo J, Kim J, Chen HS, Feuer EJ. Clustering of trend data using joinpoint regression models. *Stat Med*. 2014;33(23):4087-4103.

21. Chambers J. *Software for Data Analysis: Programming with R*. Springer Science & Business Media; 2008.

22. Nogueira RG, Abdalkader M, Qureshi MM, et al. Global impact of COVID-19 on stroke care. *Int J Stroke*. 2021;16(5):573-584.

For peer review only

23. Oxley TJ, Mocco J, Majidi S, et al. Large-Vessel Stroke as a Presenting Feature of Covid-19 in the Young. *N Engl J Med*. 2020;382(20):e60.

24. Mecca AP, Regenhardt RW, O'Connor TE, et al. Cerebroprotection by angiotensin-(1-7) in endothelin-1-induced ischaemic stroke. *Exp Physiol*. 2011;96(10):1084-1096.

25. To KF, Lo AWI. Exploring the pathogenesis of severe acute respiratory syndrome (SARS): the tissue distribution of the coronavirus (SARS-CoV) and its putative receptor, angiotensin-converting enzyme 2 (ACE2). *J Pathol*. 2004;203(3):740-743.

26. Ribes A, Vardon-Bounes F, Mémier V, et al. Thromboembolic events and Covid-19. *Adv Biol Regul*. 2020;77(100735):100735.

27. Mondal S, Quintili AL, Karamchandani K, Bose S. Thromboembolic disease in COVID-19 patients: A brief narrative review. *J Intensive Care Med*. 2020;8:70.

28. Spyropoulos AC, Goldin M, Giannis D, et al. Efficacy and Safety of Therapeutic-Dose Heparin vs Standard Prophylactic or Intermediate-Dose Heparins for Thromboprophylaxis in High-risk Hospitalized Patients With COVID-19: The HEP-COVID Randomized Clinical Trial. *JAMA Intern Med*. 2021;181(12):1612-1620.

29. Daly SR, Nguyen AV, Zhang Y, Feng D, Huang JH. The relationship between COVID-19 infection and intracranial hemorrhage: A systematic review. *Brain Hemorrhages*. 2021;2(4):141-150.

For peer review only

Figure captions:

Figure 1. Stroke of the middle cerebral artery (MCA).

Figure 2. Trends in the use of the different treatment modalities between 2016 and 2020.

Figure 3. Trends of (A) postprocedural complications and (B) neurologic complications between 2016 and 2020.

Figure 4. Map of the USA showing the distribution of patients with MCA strokes and concomitant COVID-19.

Figure 5. Illustration showing the hypercoagulable state and resulting thrombosis associated with SARS-CoV-2 infection and COVID-19.

For peer review only

For peer review only - http://bmjopen.bmj.com/site/about/guidelines.xhtml

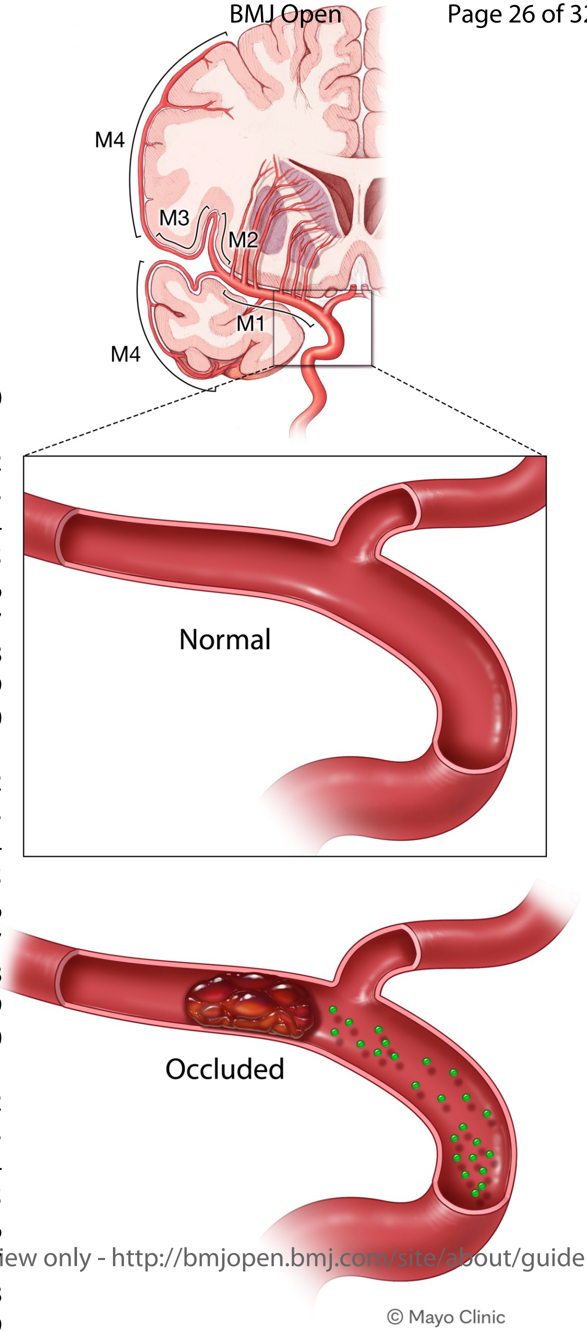

r review only - http://bmjopen.bmj.com/site/about/guideline

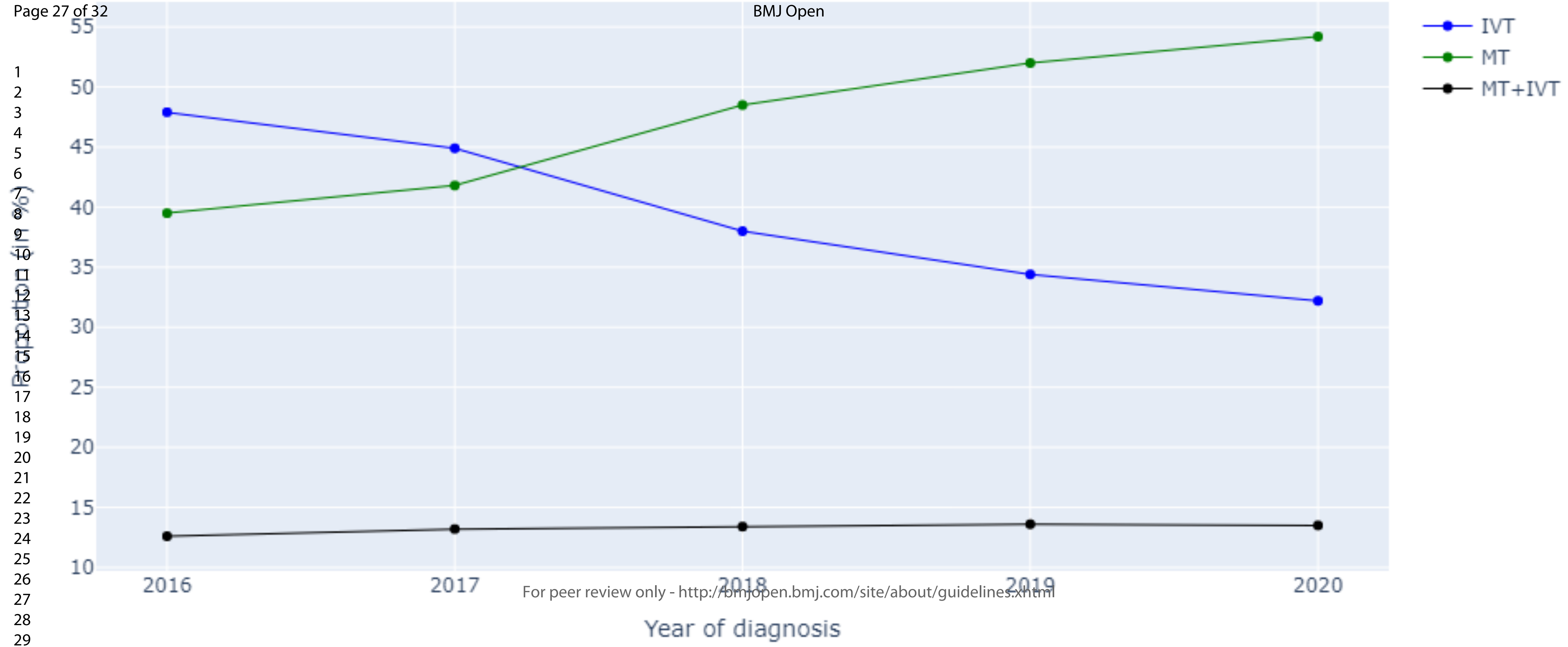
55
50
45
40
35
30
25
20
15
10
Proportion (in %)
2016
2017
2018
2019
2020
Year of diagnosis
IVT
MT
MT+IVT

A

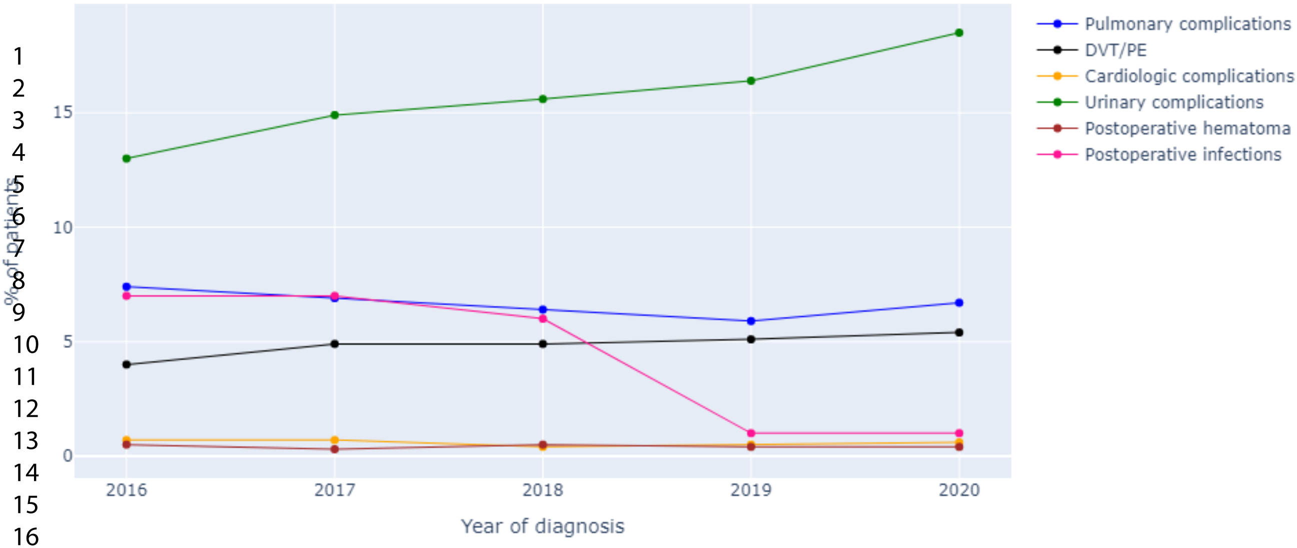

B

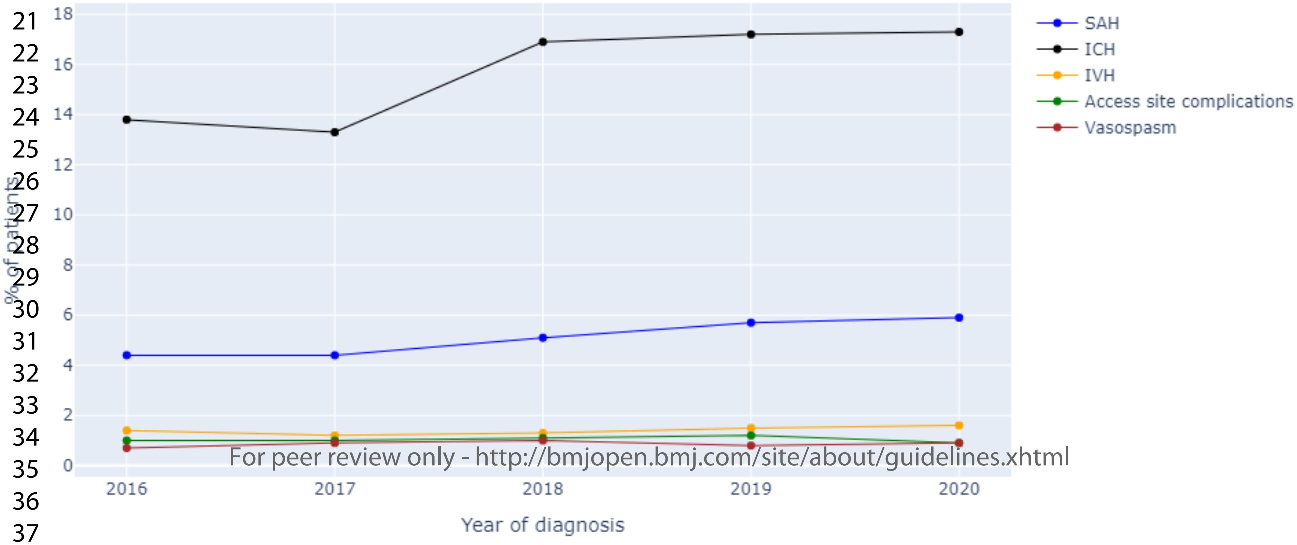

For peer review only - http://bmjopen.bmj.com/site/about/guidelines.xhtml

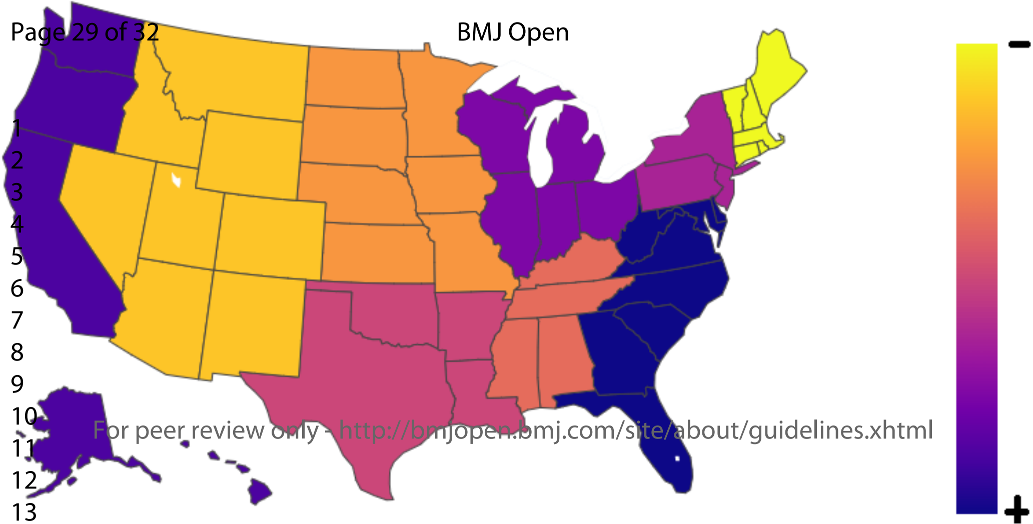

For peer review only - http://bmjopen.bmj.com/site/about/guidelines.xhtml

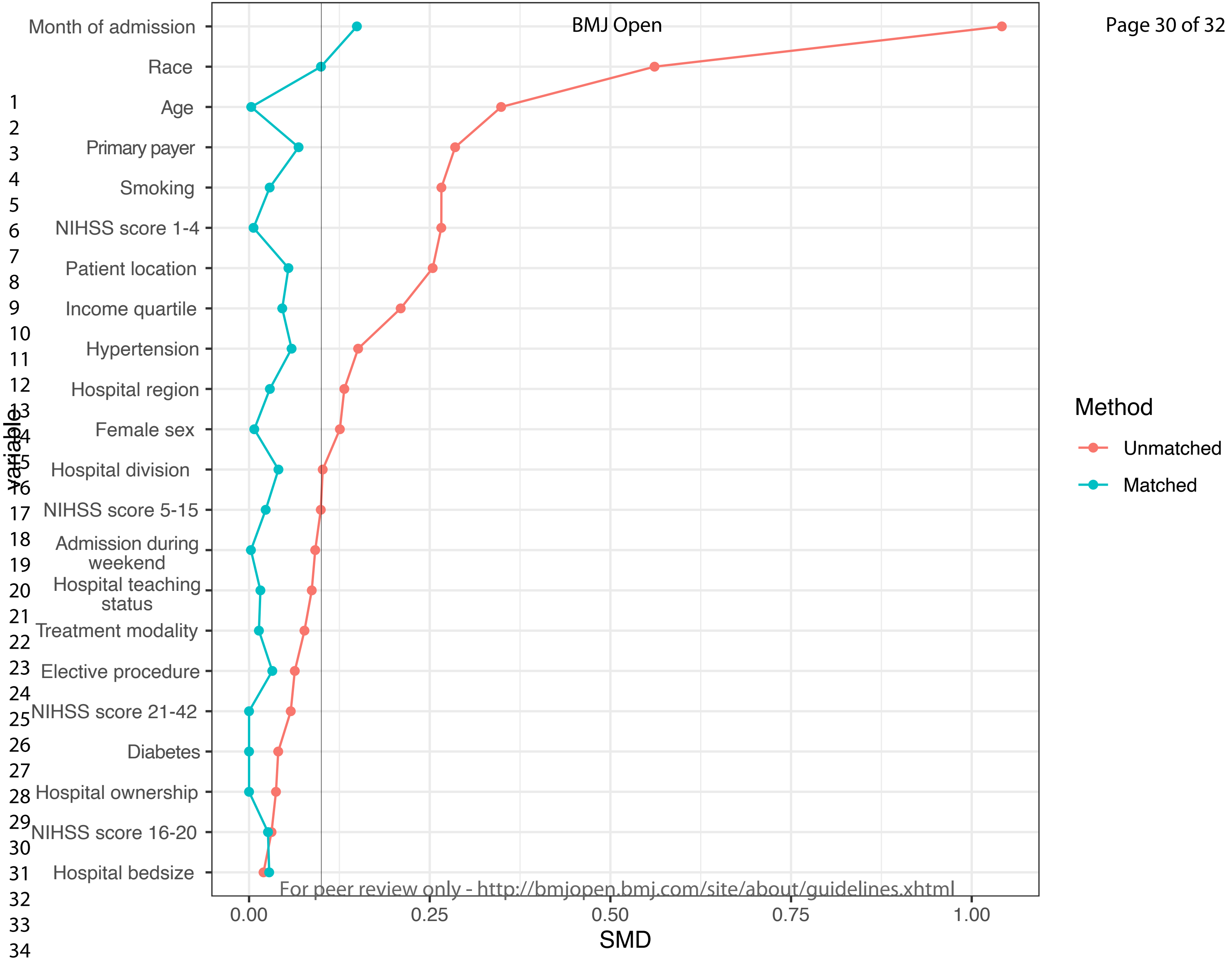
Month of admission
Race
Age
Primary payer
Smoking
NIHSS score 1-4
Patient location
Income quartile
Hypertension
Hospital region
Female sex
Hospital division
NIHSS score 5-15
Admission during weekend
Hospital teaching status
Treatment modality
Elective procedure
NIHSS score 21-42
Diabetes
Hospital ownership
NIHSS score 16-20
Hospital bedsize
variable
0.00
0.25
0.50
0.75
1.00
SMD
Method
Unmatched
Matched

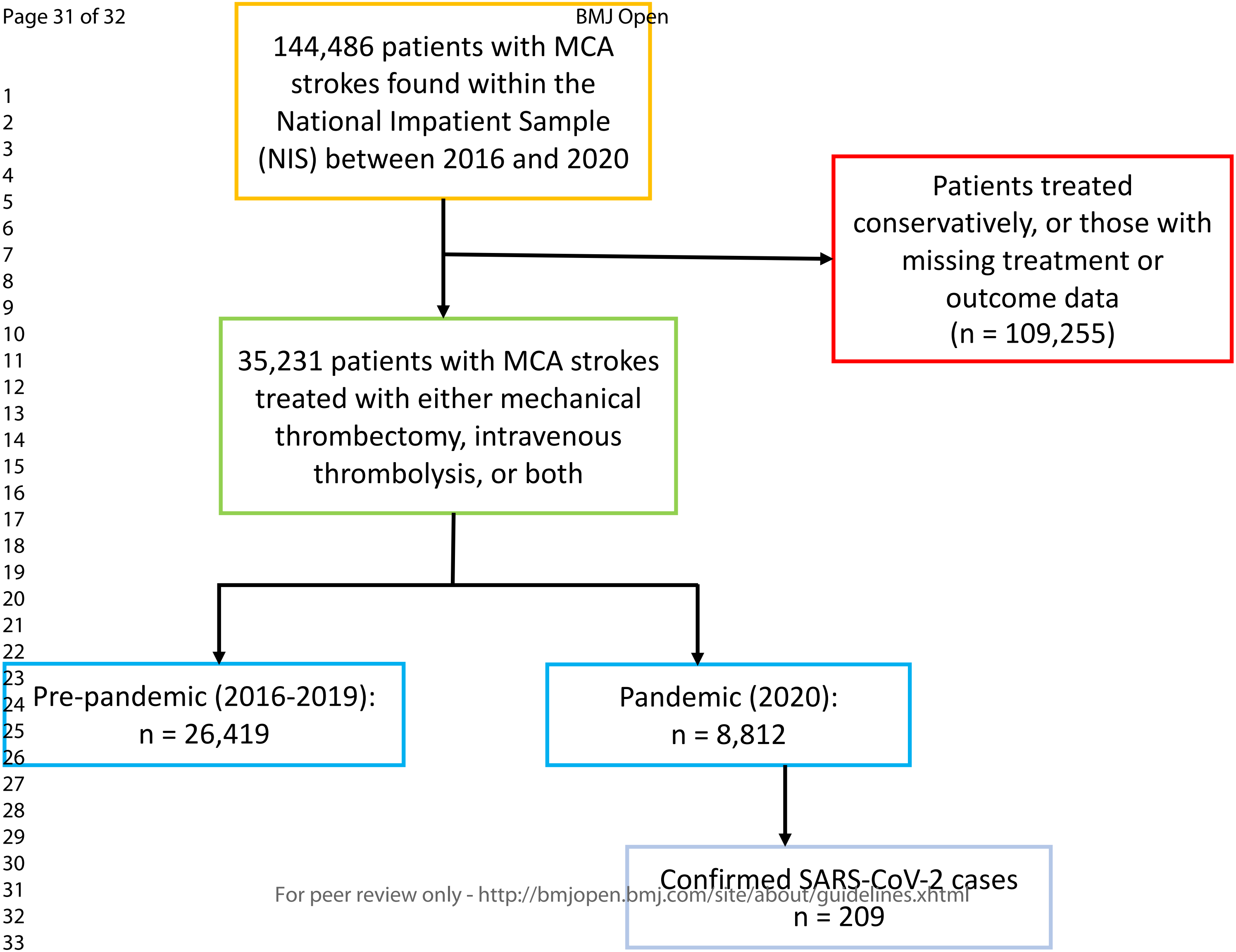
144,486 patients with MCA strokes found within the National Impatient Sample (NIS) between 2016 and 2020
Patients treated conservatively, or those with missing treatment or outcome data (n = 109,255)
35,231 patients with MCA strokes treated with either mechanical thrombectomy, intravenous thrombolysis, or both
Pre-pandemic (2016-2019): n = 26,419
Pandemic (2020): n = 8,812
Confirmed SARS-CoV-2 cases n = 209

STROBE Statement—Checklist of items that should be included in reports of ***cohort studies***

	Item No	Recommendation	Page No
**Title and abstract**	1	(*a*) Indicate the study's design with a commonly used term in the title or the abstract<br>(*b*) Provide in the abstract an informative and balanced summary of what was done and what was found	1
**Introduction**			
Background/rationale	2	Explain the scientific background and rationale for the investigation being reported	4
Objectives	3	State specific objectives, including any prespecified hypotheses	4
**Methods**			
Study design	4	Present key elements of study design early in the paper	5
Setting	5	Describe the setting, locations, and relevant dates, including periods of recruitment, exposure, follow-up, and data collection	5
Participants	6	(*a*) Give the eligibility criteria, and the sources and methods of selection of participants. Describe methods of follow-up<br>(*b*) For matched studies, give matching criteria and number of exposed and unexposed	6
Variables	7	Clearly define all outcomes, exposures, predictors, potential confounders, and effect modifiers. Give diagnostic criteria, if applicable	5-6
Data sources/ measurement	8*	For each variable of interest, give sources of data and details of methods of assessment (measurement). Describe comparability of assessment methods if there is more than one group	5-6
Bias	9	Describe any efforts to address potential sources of bias	5-6
Study size	10	Explain how the study size was arrived at	5
Quantitative variables	11	Explain how quantitative variables were handled in the analyses. If applicable, describe which groupings were chosen and why	5-6
Statistical methods	12	(*a*) Describe all statistical methods, including those used to control for confounding<br>(*b*) Describe any methods used to examine subgroups and interactions<br>(*c*) Explain how missing data were addressed<br>(*d*) If applicable, explain how loss to follow-up was addressed<br>(*e*) Describe any sensitivity analyses	5-6
**Results**			
Participants	13*	(a) Report numbers of individuals at each stage of study—eg numbers potentially eligible, examined for eligibility, confirmed eligible, included in the study, completing follow-up, and analysed<br>(b) Give reasons for non-participation at each stage<br>(c) Consider use of a flow diagram	7
Descriptive data	14*	(a) Give characteristics of study participants (eg demographic, clinical, social) and information on exposures and potential confounders<br>(b) Indicate number of participants with missing data for each variable of interest<br>(c) Summarise follow-up time (eg, average and total amount)	7
Outcome data	15*	Report numbers of outcome events or summary measures over time	10

For peer review only

For peer review only - http://bmjopen.bmj.com/site/about/guidelines.xhtml

Main results	16	(*a*) Give unadjusted estimates and, if applicable, confounder-adjusted estimates and their precision (eg, 95% confidence interval). Make clear which confounders were adjusted for and why they were included<br>(*b*) Report category boundaries when continuous variables were categorized<br>(*c*) If relevant, consider translating estimates of relative risk into absolute risk for a meaningful time period	10
Other analyses	17	Report other analyses done—eg analyses of subgroups and interactions, and sensitivity analyses	N/a
**Discussion**			
Key results	18	Summarise key results with reference to study objectives	15
Limitations	19	Discuss limitations of the study, taking into account sources of potential bias or imprecision. Discuss both direction and magnitude of any potential bias	18
Interpretation	20	Give a cautious overall interpretation of results considering objectives, limitations, multiplicity of analyses, results from similar studies, and other relevant evidence	18
Generalisability	21	Discuss the generalisability (external validity) of the study results	18
**Other information**			
Funding	22	Give the source of funding and the role of the funders for the present study and, if applicable, for the original study on which the present article is based	20

*Give information separately for exposed and unexposed groups.

Note: An Explanation and Elaboration article discusses each checklist item and gives methodological background and published examples of transparent reporting. The STROBE checklist is best used in conjunction with this article (freely available on the Web sites of PLoS Medicine at http://www.plosmedicine.org/, Annals of Internal Medicine at http://www.annals.org/, and Epidemiology at http://www.epidem.com/). Information on the STROBE Initiative is available at http://www.strobe-statement.org.

For peer review only

BMJ Open

Impact of the pandemic and concomitant COVID-19 on the management and outcomes of middle cerebral artery strokes: a nationwide registry-based study

Journal:	*BMJ Open*
Manuscript ID	bmjopen-2023-080738.R1
Article Type:	Original research
Date Submitted by the Author:	09-Jan-2024
Complete List of Authors:	Ghaith, Abdul Karim; Mayo Clinic<br>El-Hajj, Victor Gabriel; Karolinska Institute, Clinical Neuroscience<br>Atallah, Elias; Thomas Jefferson University Hospital, Neurological Surgery<br>Rios Zermeno, Jorge; Mayo Clinic in Florida<br>Ravindran, Krishnan; Mayo Clinic in Florida<br>Gharios, Maria; Karolinska Institute, Clinical Neuroscience<br>Hoang, Harry; Mayo Clinic<br>Bydon, Mohamad; Mayo Clinic<br>Ohlsson, Marcus; Karolinska Institute, Clinical Neuroscience<br>Elmi-Terander, Adrian; Karolinska Institute, Clinical Neuroscience<br>Rabih, Tawk; Mayo Clinic in Florida<br>Jabbour, Pascal; Thomas Jefferson Univ Hosp, Neurosurgery
**Primary Subject Heading**:	Neurology
Secondary Subject Heading:	Neurology, Surgery
Keywords:	COVID-19, Stroke < NEUROLOGY, NEUROLOGY

SCHOLARONE™
Manuscripts

I, the Submitting Author has the right to grant and does grant on behalf of all authors of the Work (as defined in the below author licence), an exclusive licence and/or a non-exclusive licence for contributions from authors who are: i) UK Crown employees; ii) where BMJ has agreed a CC-BY licence shall apply, and/or iii) in accordance with the terms applicable for US Federal Government officers or employees acting as part of their official duties; on a worldwide, perpetual, irrevocable, royalty-free basis to BMJ Publishing Group Ltd ("BMJ") its licensees and where the relevant Journal is co-owned by BMJ to the co-owners of the Journal, to publish the Work in this journal and any other BMJ products and to exploit all rights, as set out in our licence.

The Submitting Author accepts and understands that any supply made under these terms is made by BMJ to the Submitting Author unless you are acting as an employee on behalf of your employer or a postgraduate student of an affiliated institution which is paying any applicable article publishing charge ("APC") for Open Access articles. Where the Submitting Author wishes to make the Work available on an Open Access basis (and intends to pay the relevant APC), the terms of reuse of such Open Access shall be governed by a Creative Commons licence – details of these licences and which Creative Commons licence will apply to this Work are set out in our licence referred to above.

Other than as permitted in any relevant BMJ Author's Self Archiving Policies, I confirm this Work has not been accepted for publication elsewhere, is not being considered for publication elsewhere and does not duplicate material already published. I confirm all authors consent to publication of this Work and authorise the granting of this licence.

For peer review only

Impact of the pandemic and concomitant COVID-19 on the management and outcomes of middle cerebral artery strokes: a nationwide registry-based study

Abdul Karim Ghaith MD [1,*]; Victor Gabriel El-Hajj [1,2,*]; Elias Atallah MD [3]; Jorge Rios-Zermeno MD [4]; Krishnan Ravindran MD [4]; Maria Gharios BS [2]; Harry Hoang [1]; Mohamad Bydon MD [1]; Marcus Ohlsson MD, PhD [2]; Adrian Elmi-Terander MD, PhD [2]; Rabih Tawk MD [4]; Pascal Jabbour MD [3]

[1]Department of Neurological Surgery, Mayo Clinic, Rochester, Minnesota, USA.
[2]Department of Clinical Neuroscience, Karolinska Institutet, Stockholm, Sweden.
[3]Department of Neurological Surgery, Thomas Jefferson University Hospital, Philadelphia, USA
[4]Department of Neurological Surgery, Mayo Clinic, Jacksonville, Florida, USA.
[5]Department of Surgical Sciences, Uppsala University, Uppsala, Sweden
[6]Capio Spine Center Stockholm, Löwenströmska Hospital, Upplands Väsby, Sweden

*Contributed equally

Correspondence to:

Adrian Elmi-Terander

Capio Spine Center Stockholm, Löwenströmska Hospital, 194 02 Upplands-Väsby, Sweden (Box 2074)

Email: adrian.elmi.terander@ki.se

For peer review only

Abstract

Objectives

To investigate the impact of the COVID-19 pandemic as well as concomitant COVID-19 itself on stroke care, focusing on middle cerebral artery (MCA) territory infarctions.

Design

Registry-based study.

Setting

We used the National Inpatient Sample (NIS) database, which covers a wide range of hospitals within the United States.

Participants

The NIS was queried for patients with MCA strokes between 2016 and 2020. In total, 35,231 patients were included.

Outcome measures

Outcome measures were postprocedural complications, length of stays, in-hospital mortality, and non-routine discharge. Propensity score matching using all available baseline variables was performed to reduce confounders when comparing patients with and without concomitant COVID-19.

Results

Mechanical thrombectomy (MT) was performed in 48.4%, intravenous thrombolysis (IVT) in 38.2%, and both mechanical thrombectomy and IVT (MT-IVT) in 13.4% of patients. A gradual increase in the use of MT and an opposite decrease in the use of IVT ($p<0.001$) was detected during the study period. Overall, 25.0% of all patients were admitted for MCA strokes during the pandemic period (2020), of these 209 (2.4%) were concomitantly diagnosed with COVID-19. Patients with MCA strokes and concomitant COVID-19 were significantly younger (64.9 vs. 70.0; $p<0.001$), had significantly worse NIHSS scores, and worse outcomes in terms of length of stay (12.3 vs. 8.2; $p<0.001$), in-hospital mortality (26.3% vs. 9.8%; $p<0.001$), and non-routine discharge (84.2% vs. 76.9%; $p=0.013$), as compared with those without COVID-19. After matching, only in-hospital mortality rates remained significantly higher in patients with COVID-19 (26.7% vs. 8.5%; $p<0.001$).

For peer review only

Additionally, patients with COVID-19 had higher rates of thromboembolic (12.3% vs. 7.6%; p=0.035) and respiratory (11.3% vs. 6.6%; p=0.029) complications.

Conclusions

Among patients with MCA stroke, those with concomitant COVID-19 were significantly younger and had higher stroke severity scores. They were more likely to experience thromboembolic and respiratory complications and in-hospital mortality compared with matched controls.

For peer review only

Strengths and limitations of this study

- This study includes more than 35,000 patients admitted for strokes of the middle cerebral artery (MCA) between 2016 and 2020
- Our study attempted to discern the impact of the pandemic state itself from that of concomitant infection with SARS-CoV-2 on the management and outcomes of MCA strokes.
- This study is registry-based and limited by its hospital-based rather than population-based nature.

INTRODUCTION

Cerebral stroke is a leading cause of morbidity and mortality. In 2019 stroke was globally ranked second as the leading cause of death[1] and disability-adjusted life years (DALYs) in individuals aged above 50.[2] Recent estimates suggest that one of every four individuals are at risk of experiencing a stroke during their lifetime.[3] In addition to the associated mortality and morbidity, stroke is known to generate a significant financial burden on both individual and societal levels.[1,4]

The pandemic caused by SARS-CoV-2 (COVID-19 pandemic) hitting the world by the end of 2019, was set to impact the healthcare infrastructure in an unprecedented fashion. The COVID-19 pandemic has, directly and indirectly, caused major epidemiological changes to cerebrovascular diseases.[5] While stroke presentations significantly dropped during the pandemic,[6,7] infection with the virus was linked with an increased risk of strokes.[8–11] The pathophysiology behind this association is thought to be a hypercoagulable state, in turn, linked to abnormal platelet activation, endothelial dysfunction, and disruption of the coagulation cascade by the virus.[5,12] Other noteworthy changes during the pandemic were the rise in the prevalence of younger individuals and those with large vessel obstructions among patients with stroke.[13,14] Apart from changes to the epidemiology of stroke, several reports have identified alterations in the use of different treatment modalities during that time.[13,15,16] While many of these changes have been hypothesized to result from altered signaling pathways associated with infection with the virus, altered care-seeking behaviors, and the burden of the pandemic on healthcare infrastructures may also have played a role.[5]

However, few studies on the impact of concomitant SARS-CoV-2 infection on the management and outcomes of stroke have been conducted. Using data provided by the NIS, the aim of this study was to validate previous reports and investigate the effects of the COVID-19 pandemic and SARS-CoV-2 infection on stroke care and short-term outcomes, focusing on middle cerebral artery (MCA) territory infarctions (Figure 1).[17]

METHODS

Data source

The NIS, which is maintained by the Healthcare Cost and Utilization Project, is one of the largest inpatient care databases accessible to the public in the United States. The dataset includes approximately 7 million unweighted patient records per year, representing a 20% stratified sample of all Healthcare Cost and Utilization Project community hospitals in the

For peer review only

United States. The NIS permits extensive investigations into healthcare utilization, access, charges, quality, and outcomes and provides dependable national estimates on an annual basis. The data contains a variety of elements, including demographic characteristics, hospital and regional information, diagnoses, procedures, and discharge disposition for all patients for whom documentation exists. More information about the NIS is available at www.hcup-us.ahrq.gov. As the data was collected from a de-identified national database, Institutional Review Board (IRB) approval was not required.

Cohort selection

Patients diagnosed and treated with ischemic middle cerebral artery (MCA) stroke were identified using the International Classification of Diseases (ICD) codes “I66.0”, “I66.01”, “I66.02”, “I66.03”, “I66.09”, “I63.31”, “I633.11”, “I633.13”,“I63.319”, “I63.411”, “I63.412”, “I63.413”, “I63.419”, “I63.51”. “I63.512”, “I63.513”, “I63.513”, “I63.519” from the 10th revisions. The study considered three primary treatment modalities: mechanical thrombectomy (MT), intravenous thrombolysis (IVT), or combination of them (MT+IVT). MCA stroke patients that received no treatment were excluded from the study. Initially, 144,486 patients with MCA strokes were identified within the NIS database between 2016 to 2020. However, 64,422 patients were excluded as their treatment approach was not specified, leaving 35,231 patients for inclusion in this study. The study was performed in accordance with the STROBE guidelines.

Outcomes and variables

The following variables were extracted: age, gender, race, insurance type, smoking status, history of smoking, hypertension, diabetes, and stroke severity measured by NIH stroke severity (NIHSS) score. Hospital-related data were recorded, including household income quartile, bed size, location (rural/urban), teaching status, region (Northeast, Midwest, South, West), and control/ownership of the hospital.

The study examined several key outcomes:

Death, non-routine discharge (transfer to short-term hospital, skilled nursing or intermediate care facility, home health care, or against medical advice), length of stay (LOS in days).

For peer review only

Intervention-related complications such as access-site hemorrhage, subarachnoid hemorrhages (SAH), vasospasm, intracerebral or intraventricular hemorrhage.

Medical complications including neurological, cardiac, pulmonary, urinary, and thromboembolic events (deep venous thrombosis DVT and pulmonary embolisms PE).

Propensity score matching and statistical analysis

Using the Mann-Kendall test, trends regarding the yearly incidence of MCA ischemic stroke cases, treatment modalities, and postprocedural complications between 2016 and 2020 were evaluated.[18] When analyzing the impact of concomitant COVID-19 on management and outcomes of MCA strokes, 4:1 propensity score matching based on all available baseline variables, featured in the love plot (Supplementary Figure A), was performed, using the K-nearest method with a caliper of 0.2. All statistical analyses were conducted using Python and R software.[19]

Patient and public involvement

None.

RESULTS

Trend analysis

A total of 35,231 patients were included (Supplementary Figure B), with 13,465 receiving IVT, 17,060 undergoing mechanical thrombectomy, and 4,706 undergoing mechanical thrombectomy and IVT. Results from the trend analysis revealed that the number of patients with MCA stroke within the NIS database significantly increased between the years 2016 and 2020 ($p = 0.028$). The proportion of patients receiving IVT decreased significantly ($p = 0.027$) due to a gradual and significant increase in MT ($p = 0.027$). There were no changes in the proportion of patients undergoing both IVT and MT ($p = 0.086$; Figure 2). The complications rates remained stable ($p > 0.05$), except for a slight increase in postprocedural SAHs ($p = 0.043$), thromboembolic events ($p = 0.043$), and urinary tract infections ($p = 0.027$; Figures 3A and 3B).

For peer review only

Impact of the pandemic on management of patients with MCA stroke

There were 8,291 patients admitted for MCA stroke the year before the pandemic (2019) and 8,812 during the pandemic (2020) (Table 1). During 2020, 209 of the patients (2.4%) were concomitantly infected by SARS-CoV-2. There were no differences with respect to demographics, including age, sex, race or ethnicity, primary payer, and hospital location among the two admission periods. During the pandemic, urban, non-teaching hospitals received a larger share of patients with MCA stroke, compared to the year before (9.7% vs. 8.5%; $p = 0.012$). The choice of treatment modalities significantly differed between the two admission periods, with MT being more commonly performed during than prior to the pandemic, ($p = 0.005$). The length of hospital stay was similar between the two time periods (mean: 8.2 ± 10.0; $p = 0.239$). Postprocedural complications remained similarly prevalent r, with subarachnoid hemorrhages occurring in 5.8% of patients, vasospasm in 0.9%, thromboembolic events in 5.3%, neurological complications in 0.5%, and cardiac complications in 0.5% of patients ($p > 0.05$). However, access site hemorrhage was more common during the pre-pandemic period (1.2% vs. 0.9%; $p = 0.037$), while acute kidney injury (18.5% vs. 16.4%; $p < 0.001$), respiratory (6.7% vs. 5.9%; $p = 0.030$), and urinary complications (18.5% vs. 16.4%; $p < 0.001$) were more common during the pandemic. While the proportion of non-routine discharge (76.6%) did not significantly differ between admission periods ($p = 0.149$), there were significantly more in-hospital deaths among patients admitted for MCA stroke during the pandemic (10.2% vs. 9.0%; $p = 0.010$) (Table 2).

For peer review only

Table 1. Baseline differences between patients admitted for MCA strokes before and during the pandemic (2019 vs. 2020)

	**Total (N=17103)**	**Pre-pandemic (N=8291)**	**During the pandemic (N=8812)**	**P-value**
Female	8825 (51.6%)	4322 (52.1%)	4503 (51.1%)	0.176
Mean Age (SD)	70.0 (14.6)	70.2 (14.6)	69.8 (14.6)	0.141
Race and ethnicity				0.124
White	11315 (66.2%)	5545 (66.9%)	5770 (65.5%)	
Black	2602 (15.2%)	1232 (14.9%)	1370 (15.5%)	
Hispanic	1391 (8.1%)	657 (7.9%)	734 (8.3%)	
Asian or Pacific Islander	566 (3.3%)	286 (3.4%)	280 (3.2%)	
Native American	59 (0.3%)	32 (0.4%)	27 (0.3%)	
Other	557 (3.3%)	269 (3.2%)	288 (3.3%)	
SARS-CoV-2 positive	209 (1.2%)	n/a	209 (2.4%)	n/a
Income quartile				**0.005**
1	4718 (27.6%)	2295 (27.7%)	2423 (27.5%)	
2	4336 (25.4%)	2014 (24.3%)	2322 (26.4%)	
3	4209 (24.6%)	2130 (25.7%)	2079 (23.6%)	
4	3570 (20.9%)	1722 (20.8%)	1848 (21.0%)	
Primary payer				0.652
Medicare	10822 (63.3%)	5267 (63.5%)	5555 (63.0%)	
Medicaid	1659 (9.7%)	772 (9.3%)	887 (10.1%)	
Private insurance	3501 (20.5%)	1715 (20.7%)	1786 (20.3%)	
Self-pay	648 (3.8%)	318 (3.8%)	330 (3.7%)	
No charge	51 (0.3%)	24 (0.3%)	27 (0.3%)	
Other	398 (2.3%)	183 (2.2%)	215 (2.4%)	
Hospital region				0.439
Northeast	2975 (17.4%)	1441 (17.4%)	1534 (17.4%)	
Midwest	3496 (20.4%)	1696 (20.5%)	1800 (20.4%)	
South	6974 (40.8%)	3421 (41.3%)	3553 (40.3%)	
West	3658 (21.4%)	1733 (20.9%)	1925 (21.8%)	
Hospital location and teaching status				**0.012**
Rural	207 (1.2%)	109 (1.3%)	98 (1.1%)	
Urban non-teaching	1555 (9.1%)	702 (8.5%)	853 (9.7%)	

For peer review only

	**Total (N=17103)**	**Pre-pandemic (N=8291)**	**During the pandemic (N=8812)**	**P-value**
Urban teaching	15341 (89.7%)	7480 (90.2%)	7861 (89.2%)	
Hospital bed size				0.746
Small	1560 (9.1%)	754 (9.1%)	806 (9.1%)	
Medium	3983 (23.3%)	1952 (23.5%)	2031 (23.0%)	
Large	11560 (67.6%)	5585 (67.4%)	5975 (67.8%)	
Treatment modality				**0.005**
IVT	5693 (33.3%)	2855 (34.4%)	2838 (32.2%)	
MT	9089 (53.1%)	4309 (52.0%)	4780 (54.2%)	
MT with IVT	2321 (13.6%)	1127 (13.6%)	1194 (13.5%)	

For peer review only

Table 2. Outcome differences between patients admitted for MCA strokes before and during the pandemic (2019 vs. 2020)

	**Total (N=17103)**	**Pre-pandemic (N=8291)**	**During the pandemic (N=8812)**	**P-value**
Mean length of stay (LOS) in days (SD)	8.2 (10.0)	8.1 (9.2)	8.3 (10.8)	0.239
Subarachnoid hemorrhage	993 (5.8%)	472 (5.7%)	521 (5.9%)	0.540
Intracranial hemorrhage	2945 (17.2%)	1422 (17.2%)	1523 (17.3%)	0.819
Intraventricular hemorrhage	270 (1.6%)	128 (1.5%)	142 (1.6%)	0.723
Vasospasm	150 (0.9%)	70 (0.8%)	80 (0.9%)	0.656
Access site hemorrhage	174 (1.0%)	98 (1.2%)	76 (0.9%)	**0.037**
Hematoma	74 (0.4%)	35 (0.4%)	39 (0.4%)	0.839
Wound dehiscence	15 (0.1%)	5 (0.1%)	10 (0.1%)	0.240
Vascular catheter infection	12 (0.1%)	7 (0.1%)	5 (0.1%)	0.494
Thromboembolic events	901 (5.3%)	424 (5.1%)	477 (5.4%)	0.382
Acute kidney injury	2986 (17.5%)	1360 (16.4%)	1626 (18.5%)	**< 0.001**
Neurologic complications	94 (0.5%)	48 (0.6%)	46 (0.5%)	0.615
Respiratory complications	1084 (6.3%)	491 (5.9%)	593 (6.7%)	**0.030**
Cardiac complications	92 (0.5%)	43 (0.5%)	49 (0.6%)	0.738
Urinary complications	2986 (17.5%)	1360 (16.4%)	1626 (18.5%)	**< 0.001**
Non-routine discharge	13099 (76.6%)	6310 (76.1%)	6789 (77.1%)	0.149
In-hospital mortality	1646 (9.6%)	748 (9.0%)	898 (10.2%)	**0.010**

For peer review only

Impact of concomitant COVID-19 on management and short-term outcomes of MCA stroke

Prior to the propensity score matched analysis, 209 patients with COVID-19 (SARS-CoV-2 positive) were compared to 8,603 without COVID-19, admitted during 2020 (Table 3). A map showing the distribution of patients with MCA stroke and concomitant COVID-19 is presented (Figure 4). Patients with COVID-19 tended to be males (55% vs. 48.8%; $p = 0.073$) and were significantly younger (64.9 ± 14.4 vs. 70.0 ± 14.6; $p < 0.001$). White patients with MCA stroke were significantly less likely to present with concomitant COVID-19, opposed to all other races and ethnicities ($p < 0.001$). COVID-19 patients tended to cluster within lower income quartiles ($p = 0.065$) and were significantly more covered by Medicaid (16.35 vs. 9.9%; $p < 0.001$). There were no significant differences in hospital region, location, teaching status or number of beds ($p > 0.05$). Patients with COVID-19 had significantly worse NIHSS scores (16-42) ($p = 0.015$). The treatment of choice did not significantly differ between the groups ($p = 0.562$). The mean length of hospital stay was significantly longer among patients with COVID-19 (12.3 ± 12.0 vs. 8.2 ± 10.7; $p < 0.001$). The total hospital charges were significantly higher for patients with COVID-19 ($p < 0.001$). Medical, complications, including thromboembolic events, acute kidney failure, respiratory, and urinary infection were significantly more prevalent amongst patients with COVID-19 ($p < 0.05$). Similarly, non-routine discharges ($p < 0.001$) and in-hospital mortality ($p < 0.001$) were significantly more common in this patient group.

After propensity score matching using patient demographics, baseline characteristics and comorbidities, and stroke severity on the NIHSS scale (Supplementary Figure A), only the rate of thromboembolic events ($p = 0.035$), respiratory complications ($p = 0.029$), and in-hospital mortality ($p < 0.001$) remained significant. This suggests a direct correlation between COVID-19 and the occurrence of complications and as in-hospital mortality. This was further verified using multivariable logistic regression, which indicated that concomitant infection with SARS-CoV-2 was a significant and independent predictor of in-hospital mortality (OR: 3.5; CI95%: 2.9-4.0; $p < 0.01$). Surprisingly, COVID-19 patients were seemingly less affected by hemorrhages, in particular intracranial ones, compared to patients without COVID-19 (23.5% vs. 15.4%; $p = 0.014$) (Table 3).

For peer review only - http://bmjopen.bmj.com/site/about/guidelines.xhtml

Table 3. Differences between covid positive and negative patients admitted for MCA strokes during the pandemic year of 2020

	**Pre-matching analysis**			**Propensity score matched analysis**		
	**SARS-CoV-2 positive (N=209)**	**SARS-CoV-2 negative (N=8603)**	**P-value**	**SARS-CoV-2 positive (N=195)**	**SARS-CoV-2 negative (N=753)**	**P-value**
Female sex	94 (45.0%)	4409 (51.2%)	0.073	90 (46.2%)	346 (45.9%)	0.959
Mean age (SD)	64.9 (14.4)	70.0 (14.6)	**< 0.001**	65.4 (14.3)	65.7 (15.7)	0.773
NIHSS score			**0.015**			0.991
1-4	8 (3.8%)	918 (10.7%)		8 (4.1%)	31 (4.1%)	
5-15	66 (31.6%)	3121 (36.3%)		65 (33.3%)	249 (33.1%)	
16-20	34 (16.3%)	1302 (15.1%)		31 (15.9%)	125 (16.6%)	
21-42	42 (20.1%)	1534 (17.8%)		36 (18.5%)	148 (19.7%)	
N-miss	59 (28.2%)	1728 (20.1%)		55 (28.2%)	200 (26.6%)	
Treatment modality			0.562			0.979
IVT	65 (31.1%)	2773 (32.2%)		62 (31.8%)	245 (32.5%)	
MT	120 (57.4%)	4660 (54.2%)		110 (56.4%)	419 (55.6%)	
MT with IVT	24 (11.5%)	1170 (13.6%)		23 (11.8%)	89 (11.8%)	
**Outcomes**						
Mean length of stay (LOS) in days (SD)	12.3 (12.0)	8.2 (10.7)	**< 0.001**	10.6 (8.6)	10.4 (9.0)	0.788
Mean total charges in USD (SD)	255,920 (238,810)	192,269 (183,666)	**< 0.001**	229,414 (193,518)	231,857 (196,519)	0.878
Subarachnoid hemorrhage	16 (7.7%)	505 (5.9%)	0.280	14 (7.2%)	53 (7.0%)	0.945
Intracranial hemorrhage	33 (15.8%)	1490 (17.3%)	0.563	30 (15.4%)	177 (23.5%)	**0.014**
Intraventricular hemorrhage	2 (1.0%)	140 (1.6%)	0.447	2 (1.0%)	14 (1.9%)	0.421
Access site hemorrhage	0 (0.0%)	76 (0.9%)	0.172	0 (0.0%)	3 (0.4%)	0.377
Vasospasm	2 (1.0%)	78 (0.9%)	0.940	2 (1.0%)	11 (1.5%)	0.641
Hemorrhagic stroke	18 (8.6%)	710 (8.3%)	0.852	18 (9.2%)	83 (11.0%)	0.470
Wound dehiscence	1 (0.5%)	9 (0.1%)	0.113	1 (0.5%)	2 (0.3%)	0.584
Vascular catheter infection	0 (0.0%)	5 (0.1%)	0.727	0 (0.0%)	2 (0.3%)	0.471

For peer review only

	**Pre-matching analysis**			**Propensity score matched analysis**		
	**SARS-CoV-2 positive (N=209)**	**SARS-CoV-2 negative (N=8603)**	**P-value**	**SARS-CoV-2 positive (N=195)**	**SARS-CoV-2 negative (N=753)**	**P-value**
Thromboembolic events	25 (12.0%)	452 (5.3%)	**< 0.001**	24 (12.3%)	57 (7.6%)	**0.035**
Acute kidney injury	56 (26.8%)	1570 (18.2%)	**0.002**	49 (25.1%)	152 (20.2%)	0.132
Myocardial infarction	15 (7.2%)	487 (5.7%)	0.350	12 (6.2%)	47 (6.2%)	0.964
Neurologic complications	0 (0.0%)	46 (0.5%)	0.289	0 (0.0%)	7 (0.9%)	0.177
Respiratory complications	26 (12.4%)	567 (6.6%)	**< 0.001**	22 (11.3%)	50 (6.6%)	**0.029**
Cardiac complications	0 (0.0%)	49 (0.6%)	0.274	0 (0.0%)	5 (0.7%)	0.254
Urinary complications	56 (26.8%)	1570 (18.2%)	**0.002**	49 (25.1%)	152 (20.2%)	0.132
Non-routine discharge	176 (84.2%)	6613 (76.9%)	**0.013**	165 (84.6%)	615 (81.8%)	0.355
In-hospital mortality	55 (26.3%)	843 (9.8%)	**< 0.001**	52 (26.7%)	64 (8.5%)	**< 0.001**

DISCUSSION

In this study, 35,231 patients with MCA stroke between 2016 and 2020 were included. Results from the trend analysis revealed that the number of patients with MCA stroke within the NIS database gradually increased during the study period including the pandemic (2020). This is in opposition with previous reports showing a decrease in stroke admissions during the pandemic. Global reports have indicated a decline in the volume of stroke hospitalizations, with primary stroke centers and centers with higher COVID-19 inpatient volumes experiencing steeper declines.[5,6,15] A recovery of initial stroke hospitalization rates was not witnessed until later during the pandemic.[15] The results of this study mainly reflect the coverage of the NIS registry and may merely indicate an expansion of the database during that time, rather than an actual increase in stroke admissions. The proportion of patients receiving IVT significantly decreased during the study period (48% to 32%; p = 0.027), due to a gradual and significant increase in MT (40% to 55%; p = 0.027).

For peer review only

To our knowledge, there are no previous works contrasting the impact of concomitant infection with SARS-CoV-2 with the impact of the unique circumstances created by the pandemic itself on the management of MCA strokes.

In this study, patients with COVID-19 were younger ($p<0.001$) but had significantly worse NIHSS scores (16-42). A meta-analysis on 129 491 patients reached similar conclusions, revealing that patients who were admitted for stroke, were significantly younger, and had strokes of higher severity grades. There have been several reports highlighting the occurrence of large-vessel occlusions in young patients with COVID-19.[14,20] In one study, patients with stroke and concomitant COVID-19 were significantly younger and lacked vascular risk factors. Despite being healthier at baseline, these patients had poorer outcomes and were less likely to experience complete revascularization compared to matched controls without COVID-19.[14] The occurrence of larger stroke in this group of younger and healthier patients was hypothesized to be due to the hypercoagulable state associated with infection with SARS-CoV-2, leading to thrombosis.[5,12,21,22]

Comparing the pre-pandemic year of 2019 with the year of 2020 during which the first wave of the pandemic occurred, we found that MT was more commonly performed than IVT ($p = 0.005$). Although this discrepancy may be explained by the fact that large vessel occlusions occurred more frequently among patients with COVID-19,[5,13,23] our results did not reveal any significant difference in treatment modality between patients with and without COVID-19. It is important to note, though, that we cannot completely disregard the possibility that the observed difference might be a continuation of a pre-existing trend towards a gradual increase in the use of MT over the years.

Also, a significantly increased risk of thromboembolic events in patients with MCA strokes and concomitant COVID-19, was found in the current study. This highlights the well-established correlation between COVID-19 and the occurrence of thromboembolic events,[24,25] which lead to recommendations for prophylactic treatment of hospitalized COVID-19 patients with anticoagulation therapy.[26] Surprisingly, post-matching results indicated that 23.5% of patients without COVID-19 experienced intracranial hemorrhage compared to 15.4% of those with COVID-19 ($p = 0.014$). We hypothesize that this effect may have been the result of the hypercoagulable state associated with COVID-19.[5,12,21,22] Although previous reports seem to suggest the opposite; with increased rates of intracranial hemorrhage among patients with COVID-19, this association was mainly related to the treatment with anticoagulation therapy in this group of patients.[27] Additionally, the study period only considers the first year of the pandemic, during which

For peer review only

recommendations regarding prophylactic anticoagulation therapy still were not generalized. This, may explain the lower hemorrhage risk among these patients.[15]

Lastly, our study revealed a slightly higher in-hospital mortality rate during the first year of the pandemic as compared to the year before that (p = 0.010). This may be due to a number of factors. Firstly, the strain on the healthcare systems at that time, may have created disruptions in routine care leading to suboptimal treatment of this patient group. Additionally, the fear of contracting COVID-19 as well as the public health measures undertaken during this period may have led to delayed medical attention for stroke patients. This may have contributed to more severe cases of stroke and in turn a higher mortality. Also, concomitant infection with SARS-CoV-2 itself may have contributed to the increased death toll through larger strokes, as previously mentioned, or an increased rate of adverse events. In fact, longer length of stays (p < 0.001), risk of non-routine discharge (p = 0.013), and higher in-hospital mortality rate (p < 0.001) were all noted among patients with MCA stroke and concomitant COVID-19, which may have been the result of higher stroke severity (NIHSS scores) in this group. After adjusting for confounders, including the NIHSS scores through propensity score matching, only in-hospital mortality remained significant (p < 0.001). This direct correlation between COVID-19 and in-hospital mortality was likely the result of the increased prevalence of thromboembolic events (p = 0.035) and respiratory complications (p = 0.029) witnessed in patients with COVID-19.

Nonetheless, this study has several limitations. Primarily, the sample size of patients with concomitant Covid-19 infection was very small compared to the whole cohort of patients with MCA stroke. The data provided by the NIS is limited by its hospital-based rather than population-based nature. Additionally, as with all registry-based studies, there is a risk of reporting and coding biases, loss to follow-up, and attrition, as well as other weaknesses. The NIS also lacks clinically important endpoints, such as patient-reported, health-related quality of life, neurological, and long-term clinical outcomes, as well as granularity in terms of the cause of death and other epidemiological elements. Moreover, the registry only targets the U.S. population which limits international generalizability and calls for external validation of the findings. Although propensity score matching was performed using all baseline data available on hand, the absence of other potential confounding variables that are not captured by the NIS; including various comorbidities, limited our ability to pinpoint the true effect of COVID-19 on the outcomes of interest.

CONCLUSION

For peer review only

Among patients with MCA stroke, those with concomitant COVID-19 were significantly younger and had higher stroke severity scores on the NIHSS scale. They were more likely to experience thromboembolic complications and in-hospital mortality compared to matched controls.

For peer review only

Competing interests

No competing interests are reported by any of the authors.

Contributors

AKG, VGE: conception & design of the work, drafting of the article, critical revision, and final approval of the version to be published. EA, JRZ, KR, MG, HH: conception & design of the work, drafting of the article, and final approval of the version to be published. MB, MO, AET, RT: conception & design of the work, drafting of the article, critical revision, and final approval of the version to be published. PJ: guarantor of the review, conception & design of the work, drafting of the article, critical revision, and final approval of the version to be published.

Funding

None of the authors received funding.

Ethics approval and consent to participate

This study was performed in accordance with all ethical guidelines. The need for ethical approval was waived by the Mayo IRB due to the deidentified nature of the NIS database. Consent to participate is not applicable.

Consent for publication

Not applicable.

Data availability statement

Data are available upon reasonable request.

For peer review only

References

1 GBD 2019 Stroke Collaborators. Global, regional, and national burden of stroke and its risk factors, 1990-2019: a systematic analysis for the Global Burden of Disease Study 2019. *Lancet Neurol* 2021;**20**:795–820.

2 GBD 2019 Diseases and Injuries Collaborators. Global burden of 369 diseases and injuries in 204 countries and territories, 1990-2019: a systematic analysis for the Global Burden of Disease Study 2019. *Lancet* 2020;**396**:1204–22.

3 GBD 2016 Lifetime Risk of Stroke Collaborators, Feigin VL, Nguyen G, *et al.* Global, Regional, and Country-Specific Lifetime Risks of Stroke, 1990 and 2016. *N Engl J Med* 2018;**379**:2429–37.

4 Strilciuc S, Grad DA, Radu C, *et al.* The economic burden of stroke: a systematic review of cost of illness studies. *J Med Life* 2021;**14**:606–19.

5 Siegler JE, Abdalkader M, Michel P, *et al.* Therapeutic Trends of Cerebrovascular Disease during the COVID-19 Pandemic and Future Perspectives. *J Stroke Cerebrovasc Dis* 2022;**24**:179–88.

6 Ishaque N, Butt AJ, Kamtchum-Tatuene J, *et al.* Trends in Stroke Presentations before and during the COVID-19 Pandemic: A Meta-Analysis. *J Stroke Cerebrovasc Dis* 2022;**24**:65–78.

7 Perry R, Banaras A, Werring DJ, *et al.* What has caused the fall in stroke admissions during the COVID-19 pandemic? *J Neurol* 2020;**267**:3457–8.

8 Katsoularis I, Fonseca-Rodríguez O, Farrington P, *et al.* Risk of acute myocardial infarction and ischaemic stroke following COVID-19 in Sweden: a self-controlled case series and matched cohort study. *Lancet* 2021;**398**:599–607.

9 Tu TM, Seet CYH, Koh JS, *et al.* Acute Ischemic Stroke During the Convalescent Phase of Asymptomatic COVID-2019 Infection in Men. *JAMA Netw Open* 2021;**4**:e217498.

10 Mao L, Jin H, Wang M, *et al.* Neurologic Manifestations of Hospitalized Patients With Coronavirus Disease 2019 in Wuhan, China. *JAMA Neurol* 2020;**77**:683–90.

For peer review only

11 Merkler AE, Parikh NS, Mir S, *et al.* Risk of Ischemic Stroke in Patients With Coronavirus Disease 2019 (COVID-19) vs Patients With Influenza. *JAMA Neurol* 2020;**77**:1–7.

12 Abou-Ismail MY, Diamond A, Kapoor S, *et al.* The hypercoagulable state in COVID-19: Incidence, pathophysiology, and management. *Thromb Res* 2020;**194**:101–15.

13 Katsanos AH, Palaiodimou L, Zand R, *et al.* Changes in Stroke Hospital Care During the COVID-19 Pandemic: A Systematic Review and Meta-Analysis. *Stroke* 2021;**52**:3651–60.

14 Jabbour P, Dmytriw AA, Sweid A, *et al.* Characteristics of a COVID-19 Cohort With Large Vessel Occlusion: A Multicenter International Study. *Neurosurgery* 2022;**90**:725–33.

15 Nogueira RG, Qureshi MM, Abdalkader M, *et al.* Global Impact of COVID-19 on Stroke Care and IV Thrombolysis. *Neurology* 2021;**96**:e2824–38.

16 Katsanos AH, Palaiodimou L, Zand R, *et al.* The Impact of SARS-CoV-2 on Stroke Epidemiology and Care: A Meta-Analysis. *Ann Neurol* 2021;**89**:380–8.

17 Ng YS, Stein J, Ning M, *et al.* Comparison of clinical characteristics and functional outcomes of ischemic stroke in different vascular territories. *Stroke* 2007;**38**:2309–14.

18 Kim H-J, Luo J, Kim J, *et al.* Clustering of trend data using joinpoint regression models. *Stat Med* 2014;**33**:4087–103.

19 Chambers J. *Software for Data Analysis: Programming with R*. Springer Science & Business Media 2008.

20 Oxley TJ, Mocco J, Majidi S, *et al.* Large-Vessel Stroke as a Presenting Feature of Covid-19 in the Young. *N Engl J Med* 2020;**382**:e60.

21 Mecca AP, Regenhardt RW, O'Connor TE, *et al.* Cerebroprotection by angiotensin-(1-7) in endothelin-1-induced ischaemic stroke. *Exp Physiol* 2011;**96**:1084–96.

22 To KF, Lo AWI. Exploring the pathogenesis of severe acute respiratory syndrome (SARS): the tissue distribution of the coronavirus (SARS-CoV) and its putative receptor, angiotensin-converting enzyme 2 (ACE2). *J Pathol* 2004;**203**:740–3.

23 Nogueira RG, Abdalkader M, Qureshi MM, *et al.* Global impact of COVID-19 on stroke care. *Int J Stroke* 2021;**16**:573–84.

For peer review only

24 Ribes A, Vardon-Bounes F, Mémier V, *et al.* Thromboembolic events and Covid-19. *Adv Biol Regul* 2020;**77**:100735.

25 Mondal S, Quintili AL, Karamchandani K, *et al.* Thromboembolic disease in COVID-19 patients: A brief narrative review. *J Intensive Care Med* 2020;**8**:70.

26 Spyropoulos AC, Goldin M, Giannis D, *et al.* Efficacy and Safety of Therapeutic-Dose Heparin vs Standard Prophylactic or Intermediate-Dose Heparins for Thromboprophylaxis in High-risk Hospitalized Patients With COVID-19: The HEP-COVID Randomized Clinical Trial. *JAMA Intern Med* 2021;**181**:1612–20.

27 Daly SR, Nguyen AV, Zhang Y, *et al.* The relationship between COVID-19 infection and intracranial hemorrhage: A systematic review. *Brain Hemorrhages* 2021;**2**:141–50.

For peer review only

Figure titles:

Figure 1. Stroke of the middle cerebral artery (MCA)

Figure 2. Trends in the use of the different treatment modalities between 2016 and 2020

Figure 3. Trends of (A) postprocedural complications and (B) neurologic complications between 2016 and 2020

Figure 4. Map of the USA showing the distribution of patients with MCA strokes and concomitant COVID-19

For peer review only

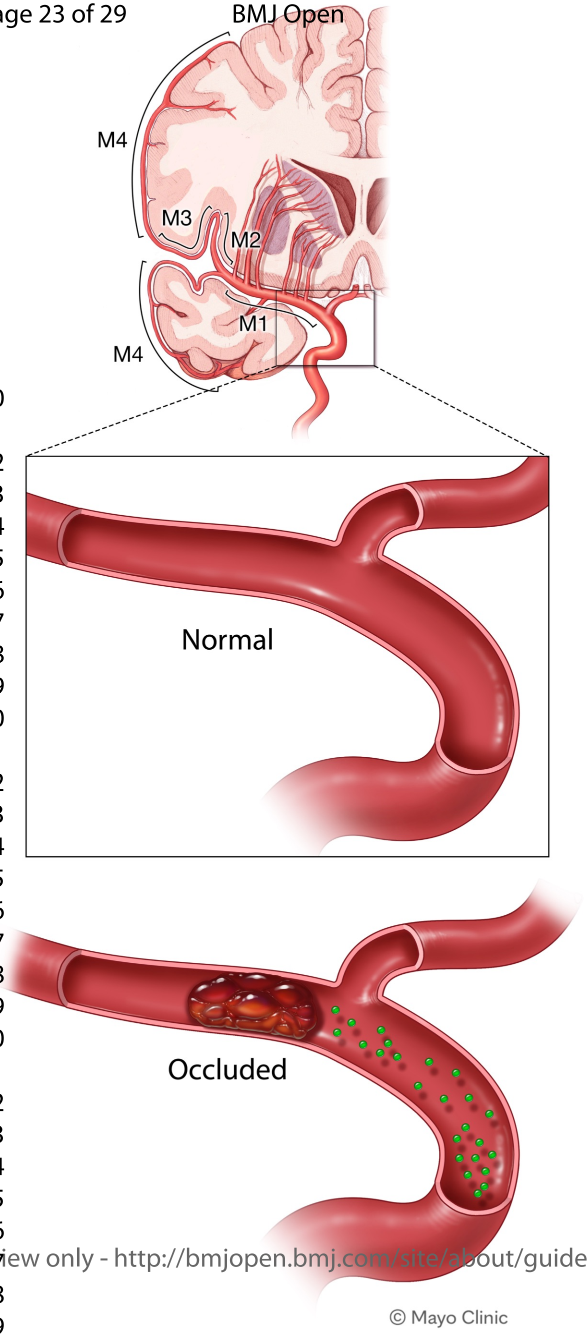

r review only - http://bmjopen.bmj.com/site/about/guideline

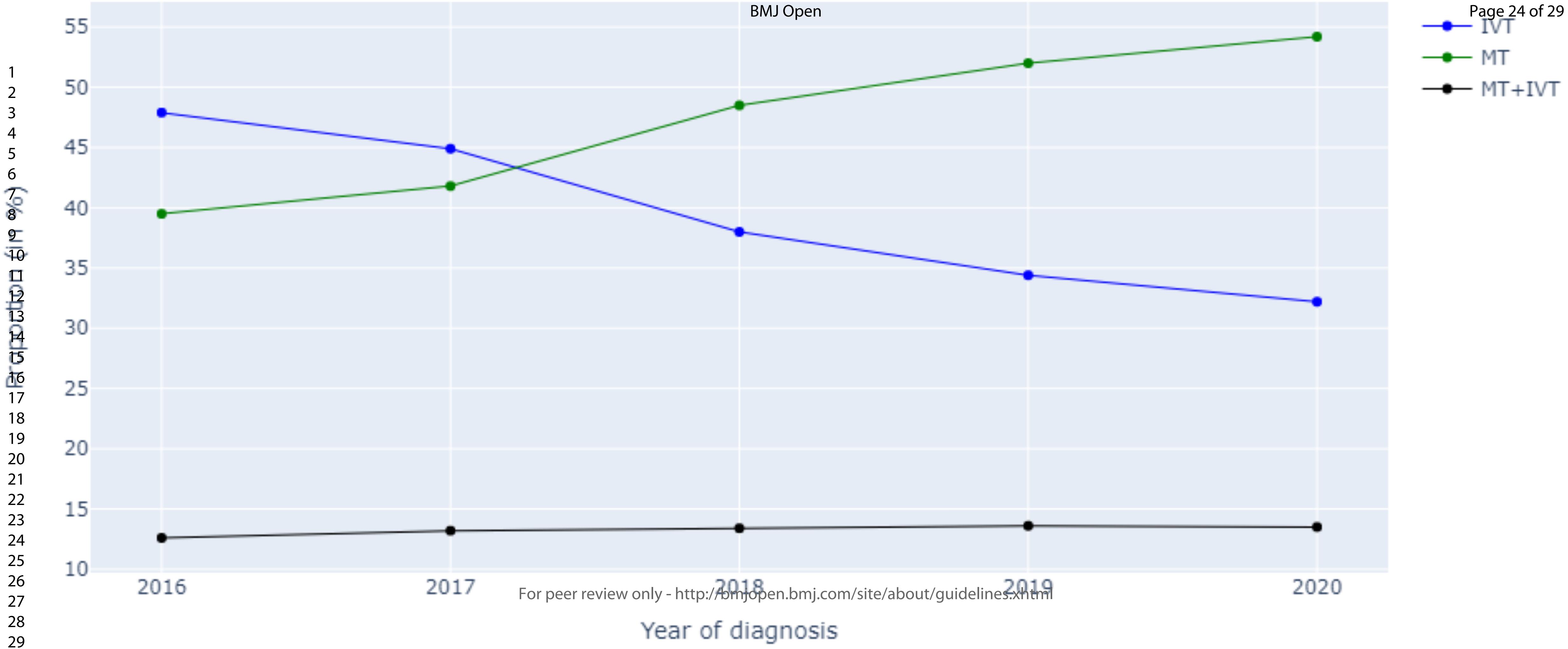
IVT
MT
MT+IVT
Proportion (in %)
55
50
45
40
35
30
25
20
15
10
2016
2017
2018
2019
2020
Year of diagnosis

A

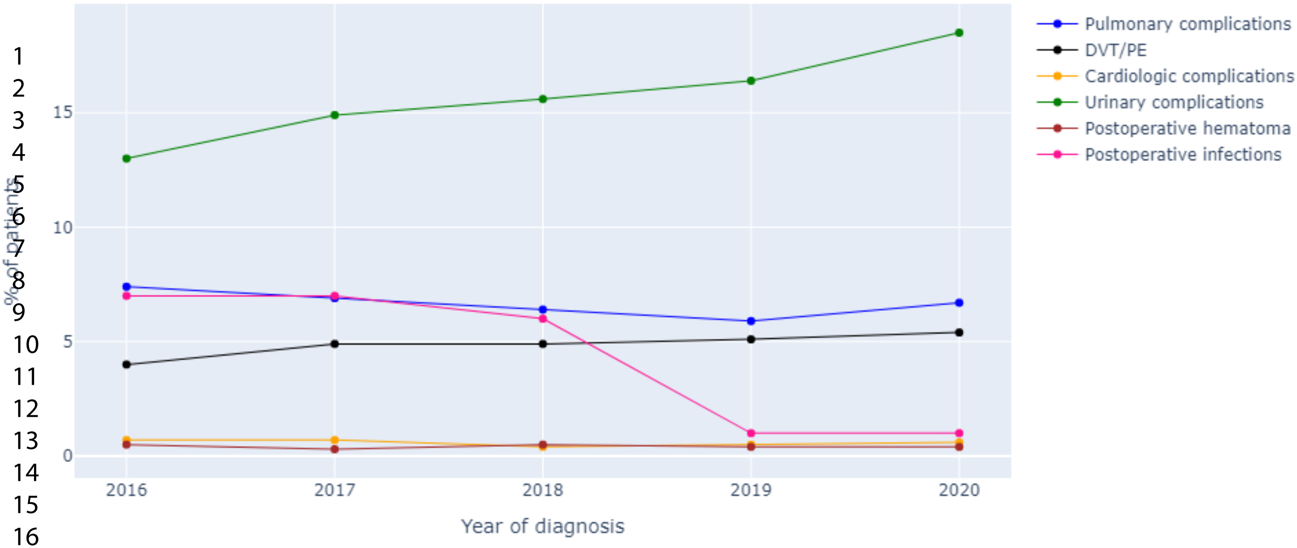
Pulmonary complications
DVT/PE
Cardiologic complications
Urinary complications
Postoperative hematoma
Postoperative infections
% of patients
Year of diagnosis
0
5
10
15
2016
2017
2018
2019
2020

B

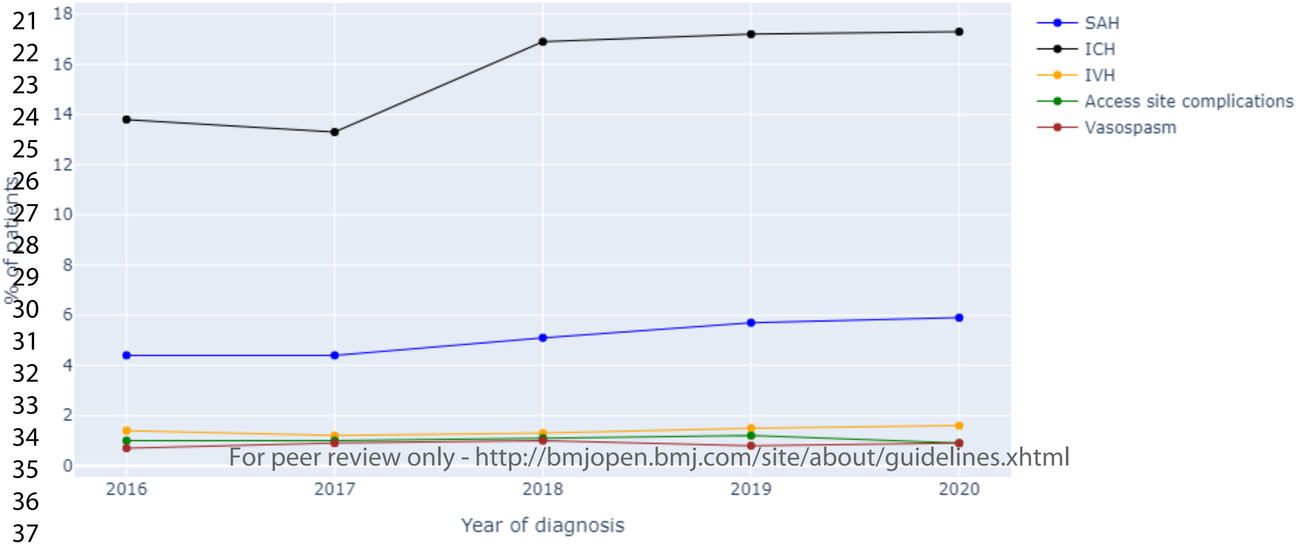
SAH
ICH
IVH
Access site complications
Vasospasm
% of patients
Year of diagnosis
0
2
4
6
8
10
12
14
16
18
2016
2017
2018
2019
2020

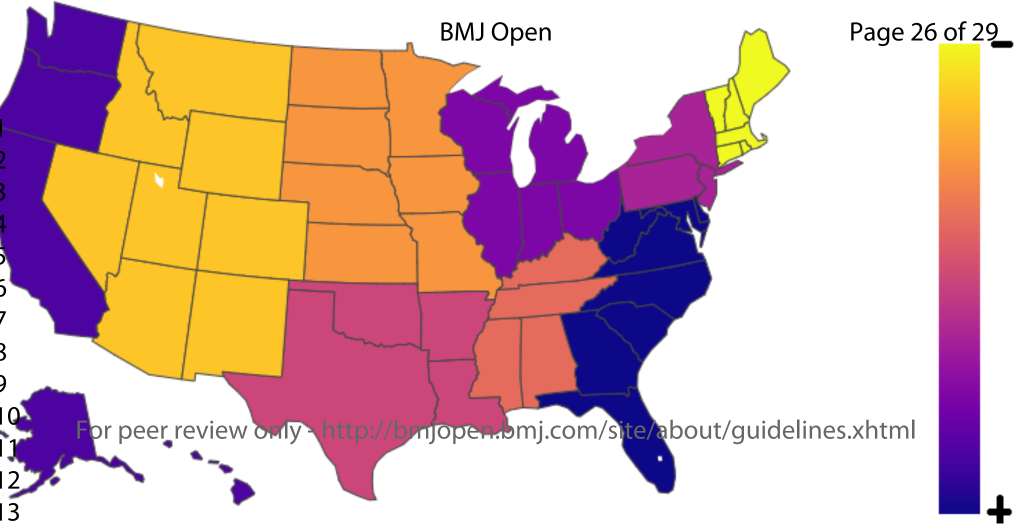

For peer review only - http://bmjopen.bmj.com/site/about/guidelines.xhtml

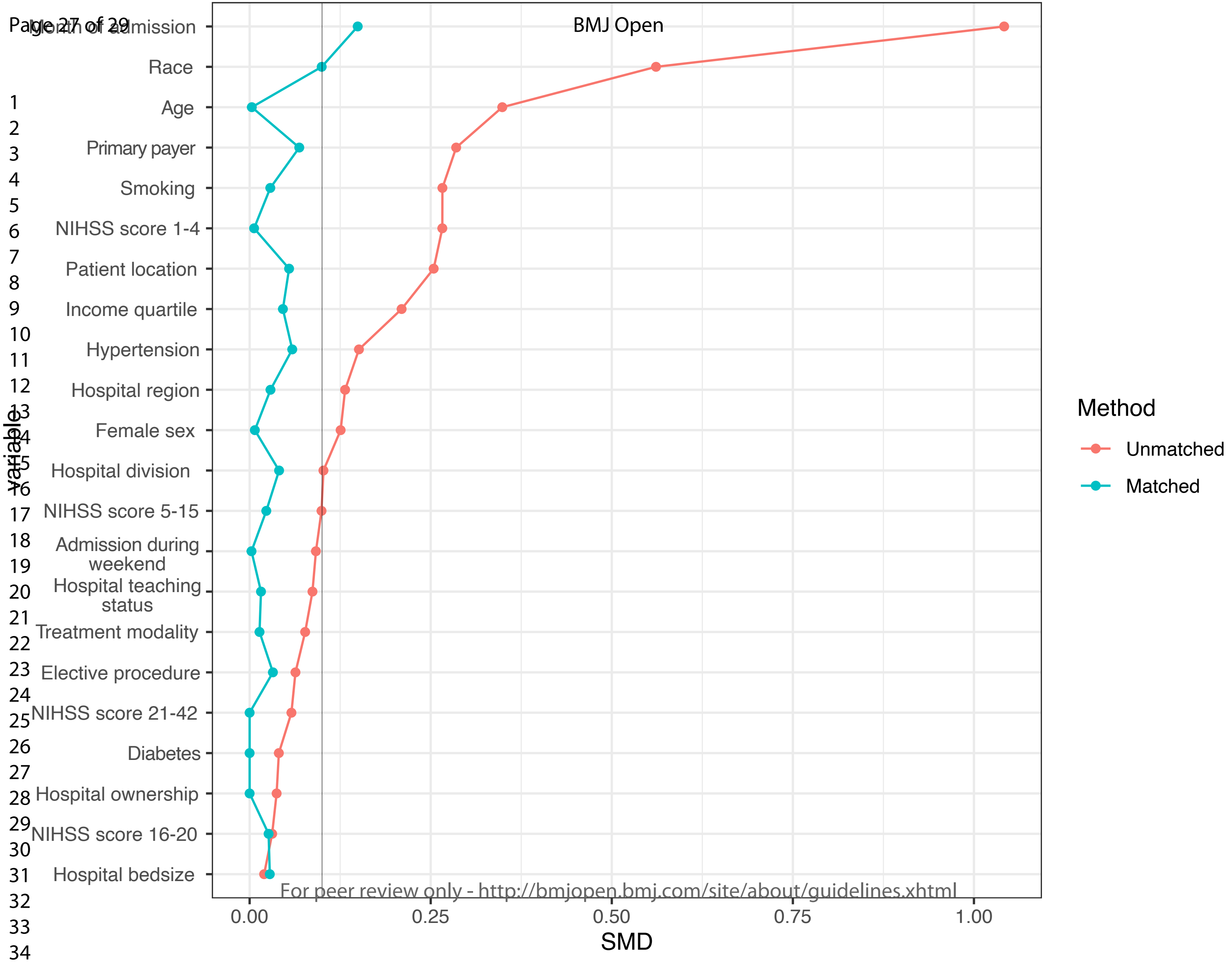
Month of admission
Race
Age
Primary payer
Smoking
NIHSS score 1-4
Patient location
Income quartile
Hypertension
Hospital region
Female sex
Hospital division
NIHSS score 5-15
Admission during weekend
Hospital teaching status
Treatment modality
Elective procedure
NIHSS score 21-42
Diabetes
Hospital ownership
NIHSS score 16-20
Hospital bedsize
variable
0.00
0.25
0.50
0.75
1.00
SMD
Method
Unmatched
Matched

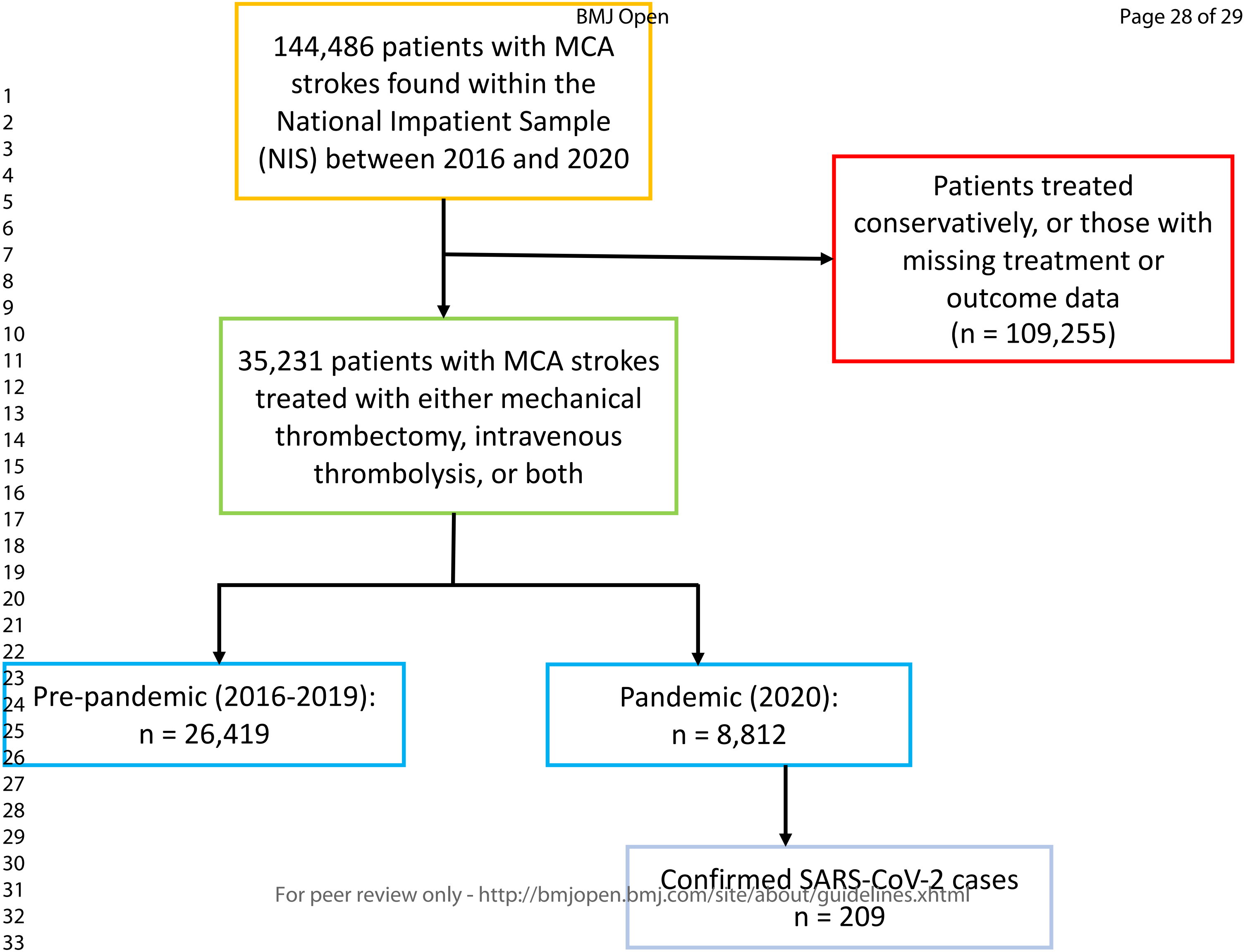

For peer review only - http://bmjopen.bmj.com/site/about/guidelines.xhtml

STROBE Statement—Checklist of items that should be included in reports of ***cohort studies***

	Item No	Recommendation	Page No
**Title and abstract**	1	(*a*) Indicate the study's design with a commonly used term in the title or the abstract<br>(*b*) Provide in the abstract an informative and balanced summary of what was done and what was found	1
**Introduction**			
Background/rationale	2	Explain the scientific background and rationale for the investigation being reported	4
Objectives	3	State specific objectives, including any prespecified hypotheses	4
**Methods**			
Study design	4	Present key elements of study design early in the paper	5
Setting	5	Describe the setting, locations, and relevant dates, including periods of recruitment, exposure, follow-up, and data collection	5
Participants	6	(*a*) Give the eligibility criteria, and the sources and methods of selection of participants. Describe methods of follow-up<br>(*b*) For matched studies, give matching criteria and number of exposed and unexposed	6
Variables	7	Clearly define all outcomes, exposures, predictors, potential confounders, and effect modifiers. Give diagnostic criteria, if applicable	5-6
Data sources/ measurement	8*	For each variable of interest, give sources of data and details of methods of assessment (measurement). Describe comparability of assessment methods if there is more than one group	5-6
Bias	9	Describe any efforts to address potential sources of bias	5-6
Study size	10	Explain how the study size was arrived at	5
Quantitative variables	11	Explain how quantitative variables were handled in the analyses. If applicable, describe which groupings were chosen and why	5-6
Statistical methods	12	(*a*) Describe all statistical methods, including those used to control for confounding<br>(*b*) Describe any methods used to examine subgroups and interactions<br>(*c*) Explain how missing data were addressed<br>(*d*) If applicable, explain how loss to follow-up was addressed<br>(*e*) Describe any sensitivity analyses	5-6
**Results**			
Participants	13*	(a) Report numbers of individuals at each stage of study—eg numbers potentially eligible, examined for eligibility, confirmed eligible, included in the study, completing follow-up, and analysed<br>(b) Give reasons for non-participation at each stage<br>(c) Consider use of a flow diagram	7
Descriptive data	14*	(a) Give characteristics of study participants (eg demographic, clinical, social) and information on exposures and potential confounders<br>(b) Indicate number of participants with missing data for each variable of interest<br>(c) Summarise follow-up time (eg, average and total amount)	7
Outcome data	15*	Report numbers of outcome events or summary measures over time	10

For peer review only

For peer review only - http://bmjopen.bmj.com/site/about/guidelines.xhtml

Main results	16	(*a*) Give unadjusted estimates and, if applicable, confounder-adjusted estimates and their precision (eg, 95% confidence interval). Make clear which confounders were adjusted for and why they were included<br>(*b*) Report category boundaries when continuous variables were categorized<br>(*c*) If relevant, consider translating estimates of relative risk into absolute risk for a meaningful time period	10
Other analyses	17	Report other analyses done—eg analyses of subgroups and interactions, and sensitivity analyses	N/a
**Discussion**			
Key results	18	Summarise key results with reference to study objectives	15
Limitations	19	Discuss limitations of the study, taking into account sources of potential bias or imprecision. Discuss both direction and magnitude of any potential bias	18
Interpretation	20	Give a cautious overall interpretation of results considering objectives, limitations, multiplicity of analyses, results from similar studies, and other relevant evidence	18
Generalisability	21	Discuss the generalisability (external validity) of the study results	18
**Other information**			
Funding	22	Give the source of funding and the role of the funders for the present study and, if applicable, for the original study on which the present article is based	20

*Give information separately for exposed and unexposed groups.

Note: An Explanation and Elaboration article discusses each checklist item and gives methodological background and published examples of transparent reporting. The STROBE checklist is best used in conjunction with this article (freely available on the Web sites of PLoS Medicine at http://www.plosmedicine.org/, Annals of Internal Medicine at http://www.annals.org/, and Epidemiology at http://www.epidem.com/). Information on the STROBE Initiative is available at http://www.strobe-statement.org.

For peer review only

For peer review only - http://bmjopen.bmj.com/site/about/guidelines.xhtml
